# Vasculoprotective Effects of Sodium-Glucose Co-Transporter Inhibitors in Non-Diabetic Experimental Settings: A Narrative Review

**DOI:** 10.3390/ijms27062573

**Published:** 2026-03-11

**Authors:** Darius G. Buriman, Lavinia Noveanu, Adina V. Furdui-Lința, Horea B. Feier, Antigone Lazou, Attila Kiss, Bruno K. Podesser, Maria D. Dănilă, Adrian Sturza, Danina M. Muntean

**Affiliations:** 1 Doctoral School Medicine, “Victor Babeș” University of Medicine and Pharmacy of Timișoara, E. Murgu Sq., No. 2, 300041 Timișoara, Romania; darius.buriman@umft.ro (D.G.B.); adina.linta@umft.ro (A.V.F.-L.); 2 Department III Functional Sciences—Pathophysiology, “Victor Babeș” University of Medicine and Pharmacy of Timișoara, E. Murgu Sq. No. 2, 300041 Timișoara, Romania; noveanu@umft.ro (L.N.); daninamuntean@umft.ro (D.M.M.); 3 Center for Translational Research and Systems Medicine, “Victor Babeș” University of Medicine and Pharmacy of Timișoara, E. Murgu Sq. No. 2, 300041 Timișoara, Romania; 4 Department of Cardiology—Clinic of Cardiovascular Surgery, “Victor Babeș” University of Medicine and Pharmacy of Timișoara, 300041 Timișoara, Romania; horea.feier@umft.ro; 5 Research Center of the Institute for Cardiovascular Diseases of Timisoara, G. Adam Str. No. 13A, 300310 Timișoara, Romania; 6 School of Biology, Aristotle University of Thessaloniki, 54124 Thessaloniki, Greece; lazou@bio.auth.gr; 7 Center for Biomedical Research and Translational Surgery, Medical University of Vienna, 1090 Vienna, Austria; attila.kiss@meduniwien.ac.at (A.K.); bruno.podesser@meduniwien.ac.at (B.K.P.)

**Keywords:** sodium-glucose cotransporter inhibitors, off-target effects, vascular protective mechanisms, non-diabetic settings, animal models, cell lines, human samples

## Abstract

Sodium-glucose co-transporter (SGLT) inhibitors are a novel class of glucose-lowering drugs with beneficial pleiotropic effects that have been widely investigated in the past decade in several experimental models and patients in the absence of diabetes. There are two types of transporters: the SGLT1 isoform that is distributed across a broad range of tissues, including the cardiovascular system, and the SGLT2 isoform, which is mostly expressed in renal proximal tubular cells. It is known that inflammation and oxidative stress are key contributors to vascular damage and the progression of atherosclerosis. SGLT inhibitors have demonstrated multiple benefits that contribute to improved vascular health, including alleviation of endothelial function, anti-inflammatory and antioxidative effects, and mitigation of arterial stiffness, all contributing to blood pressure decrease. An increasing body of research has tackled the molecular and cellular mechanisms of their chronic and, more recently, acute cardiovascular beneficial effects. This narrative review specifically delves into the direct vasculoprotective effects of SGLT2 and dual SGLT1/2 inhibitors, summarizing their off-target mechanisms described in various experimental settings (animal models, animal and human cell lines/samples).

## 1. Introduction

Glucose and sodium transport across cell membranes is greatly reliant on sodium-glucose co-transporters (SGLTs), a family of six protein isoforms [1], among which SGLT1 and SGLT2 are the most widely studied [2]. SGLT1 is primarily localized on the brush border of the small intestine. SGLT2 is predominantly expressed in the S1 and S2 segments of the renal proximal tubules, where it plays a pivotal role in the rapid reabsorption of glucose, and is the most extensively characterized transporter [3]. Human smooth muscle cells and endothelial cells also express the SGLT2 protein, albeit to a much lesser extent than renal tubular cells [4], while atherosclerotic plaques isolated from diabetic patients display higher levels of SGLT2 versus those obtained from non-diabetic individuals. Consequently, these cotransporters play a key role in vascular function and regulation.

Currently, SGLT2 inhibitors (SGLT2i) are the mainstay of treatment for patients with type 2 diabetes mellitus (T2DM) [5] owing to their effectiveness in plasma glucose level management and, also, to other beneficial effects that extend beyond glucose control. Indeed, irrespective of the patient diabetic status, SGLT2 inhibitors display unequivocal cardiac and renal protective effects (reviewed in refs. [6,7]) that warranted their inclusion in the most recent heart failure and chronic kidney disease guidelines for clinical practice [8,9]. A variety of both acute and chronic, glucose-independent effects are especially relevant to endothelial function. Endothelial dysfunction is now recognized as a central pathophysiological link between cardiovascular diseases and chronic kidney disease (CKD), even in the absence of diabetes [10]. Recent data suggest that SGLT2i exert direct, off-target effects on the endothelial cells and vascular smooth muscle, independent of glycemic control. For example, empagliflozin (EMPA) has been demonstrated to attenuate endothelial activation, lower production of adhesion molecules and pro-inflammatory cytokines, and consequently alleviate vascular inflammation [11]. Similarly, dapagliflozin (DAPA) increased endothelium-dependent vasorelaxation by enhancing nitric oxide (NO) bioavailability and reducing oxidative stress [12]. In several preclinical models, treatment with EMPA or DAPA downregulated the NLRP3 inflammasome and NF-κB inflammatory pathway, suppressed the secretion of IL-6 and TNF-α, indicating that SGLT2 inhibition provided a conserved anti-inflammatory effect that underlies both cardio- and neuroprotection [13]. Besides modulating endothelial NO signaling and reactive oxygen species (ROS) generation, SGLT2 inhibitors also impacted mitochondrial and lysosomal functions [14]. In experimental settings, SGLT2 inhibition prevented mitochondrial dysfunction and oxidative damage by inhibiting the Na^+^/H^+^ exchanger (NHE1), which lowers intracellular sodium and calcium in vascular tissues. At the same time, endothelial autophagy and resistance to ischemia or inflammatory insults are enhanced via the AMPK and sirtuin 1 (SIRT1) signaling activity [15]. Taken together, the evidence suggests that the endothelium is a critical off-target site of SGLT2 inhibition, with pleiotropic effects extending far beyond glycemic modulation in most non-communicable diseases.

Atherosclerosis (ATS), the primary underlying pathology of coronary artery disease, stroke and peripheral artery disease, is nowadays recognized as a slowly progressing, sterile, low-grade inflammatory condition [16], ultimately leading to heart failure [17]. A myriad of preclinical studies supported the glycemia-independent anti-atherosclerotic effects of SGLT2i that increase atheroma plaque stability and reduce arterial stiffness, vascular inflammation and oxidative stress, thus diminishing the cardiovascular risk [12,18,19,20,21,22,23,24,25,26,27]. Heart failure is no longer viewed solely as a cardiac disease but also as a systemic vascular disorder. Arterial stiffness and microvascular dysfunction critically contribute to increased ventricular afterload and impaired myocardial perfusion, respectively, thereby promoting disease progression, irrespective of diabetic status [28].

Moreover, there has been a growing focus on vascular aging and endothelial senescence as central drivers of cardiovascular disease progression [29]. Several lines of evidence suggest that SGLT2i may modulate multiple mechanisms of aging, including mitochondrial dysfunction, inflammasome activation, impaired autophagic flux, and altered cellular energy sensing [30]. These pathomechanisms are equally relevant in vascular aging where endothelial dysfunction and chronic low-grade inflammation often precede the overt metabolic disease and contribute to the cardiovascular risk [31].

Despite the rapidly expanding clinical proofs of the cardio-reno-metabolic benefits of SGLT2i, the precise mechanisms underlying their vasculoprotective effects in non-diabetic conditions remain incompletely understood. The present narrative review aims to summarize the experimental mechanistic evidence supporting the vascular protection, particularly the alleviation of inflammation, oxidative stress, and mitochondrial and endothelial dysfunction. By highlighting the glycemia-independent actions of the main SGLT2i, DAPA, EMPA and canagliflozin (CANA) and the main dual SGLT1/2 inhibitor, sotagliflozin (SOTA), on the vessel walls and the inflammatory cells infiltrating them, this review seeks to clarify the known molecular pathways that underlie the beneficial effects in non-diabetic settings, thus underscoring their unique profile as protective agents beyond their well-established antidiabetic role.

### Data Sources

We searched the PubMed and Google Scholar databases using the following terms (used as single keywords or searched in combination): “sodium-glucose cotransporter inhibitors”; “off-target effects”, “vasculoprotective mechanisms”, ‘vascular protection”, “pathomechanisms”, “endothelial dysfunction”, ‘mitochondrial dysfunction”, “anti-oxidant effects”, “anti-inflammatory effects”, “animal models”, “cell lines”, “human samples”. The search was conducted without date restrictions up to December 2025. In order to find more relevant studies, we additionally manually reviewed the reference lists of all qualifying studies (original publications, narrative/systematic reviews, meta-analyses).

## 2. Glycemia-Independent Vasculoprotective Mechanisms of SGLT2 Inhibitors

### 2.1. Alleviation of Inflammation

Inflammation represents a central component of ATS, and SGLT2 inhibitors have been consistently shown to exert anti-inflammatory effects that extend beyond glycemic control. By mitigating endothelial dysfunction, gliflozins decrease the endothelial expression of adhesion molecules and pro-inflammatory mediators, ultimately reducing atherosclerotic plaque development. In addition, SGLT2i attenuated lymphocyte and monocyte infiltration into the vascular wall and promoted macrophage polarization toward the anti-inflammatory M2 phenotype, while suppressing the M1-driven secretion of major pro-inflammatory markers, thus limiting the formation of foam cells [32]. The molecular mechanisms underlying these anti-atherogenic effects are complex and not yet fully elucidated but appear to involve suppression of NLRP3 inflammasome activation, downregulation of multiple inflammatory signaling pathways (NF-κB, JAK/STAT, mTOR), and activation of AMPK- and SIRT1-dependent signaling cascades [11].

#### 2.1.1. Dapagliflozin

A growing body of evidence indicates that DAPA exerts direct cellular effects on endothelial cells, immune cells, and platelets, modulating key molecular pathways implicated in atherogenesis, vascular inflammation, oxidative stress, and thrombosis. Importantly, many of these actions appear to occur independently of ambient glucose levels, supporting a pleiotropic mode of action that extends beyond glucose lowering.

As such, DAPA has been extensively investigated in experimental models for its anti-inflammatory and vasculoprotective properties in non-diabetic settings. The anti-inflammatory effects of DAPA were described both at the vascular as well as extra-vascular level.

Abdollahi et al. aimed to assess the effects of DAPA on two cell types relevant for atherogenesis, namely macrophages and human umbilical vein endothelial cells (HUVECs), which were challenged with lipopolysaccharide (LPS) under either normal or high glucose conditions. Independently of glucose concentration, DAPA attenuated LPS-induced overexpression of TLR-4, NF-κB phosphorylation and pro-inflammatory cytokines’ release and elevated the level of the anti-inflammatory miR-146a along with a substantial polarization towards the M2 macrophage phenotype [33]. Another in vitro study performed on HUVECs revealed that DAPA downregulates the TNF-α- or hyperglycemia-induced overexpression of ICAM-1 (intercellular adhesion molecule-1), VCAM-1 (vascular cell adhesion molecule-1), PAI-1 (plasminogen activator inhibitor type 1) and NF-κB [34].

Faridvand et al. reported that HUVECs treated with DAPA prior to the establishment of a high glucose pericellular environment displayed a significant up-regulation of the AMPK/SIRT1 pathway, leading to improved cellular viability with reduced generation of IL-6, TNF-α and ROS [35]. Since cardiovascular diseases are known to promote a dysregulated glucose metabolism via their associated inflammatory profile or the drugs used for their therapy [36,37], this latter study [35] points to the DAPA-induced protection at the endothelial level in the event of hyperglycemia development.

In another recent study carried out in human aortic endothelial cells (HAECs), DAPA counteracted the TNF-α-induced pro-oxidant and pro-inflammatory effects [38]. At the molecular level, DAPA alleviated oxidative stress via the NRF2 pathway, stimulated the pro-survival PI3K/AKT pathway, partially reduced endoplasmic reticulum stress and decreased the expression of NLRP3, NF-κB, IL-1β, IL-6, TNF-α and that of adhesion molecules ICAM-1 and VCAM-1. Moreover, as previously noted, in this inflammatory environment the protein expressions of SGLT2 and NHE1 were increased, while DAPA treatment led to their significant reduction, a fact which points to DAPA’s potential for endothelial glucotoxicity limitation by decreasing glucose uptake at the cellular level [38].

Importantly, NHE1 has emerged as a potential contributor to the aggravation of atherogenesis. For instance, low shear stress promotes AMPK dephosphorylation, which acts via NHE1 activation to promote endothelial glycocalyx degradation, vascular macrophage recruitment and inflammation [39]. Spigoni et al. revealed that EMPA and DAPA mitigate stearate-induced lipotoxicity in myeloid angiogenic cells (endothelial progenitor cells) and platelets isolated from healthy subjects via NHE inhibition. The authors noted that SGLT2i treatment was associated with reduced platelet activation and diminished myeloid angiogenic cell oxidative stress and production of inflammatory markers (IL-1β, TNF-α), respectively [40]. Additionally, Lescano et al. demonstrated the inhibition of human platelet activation by DAPA, EMPA and CANA, an effect potentiated by NO and prostacyclin [41]. This highlights a possible plaque stabilization and thrombosis inhibition role, partially explaining the ability of EMPA and DAPA to reduce the rate of major adverse cardiovascular events in large outcome trials.

In a non-diabetic murine model of acute lung injury, DAPA mitigated oxidative stress and elicited AMPK phosphorylation along with a significant reduction in NF-κB, NLRP3 inflammasome and caspase-1 levels and activity [42].

Using normoglycemic rabbits fed a high-cholesterol diet, Lee et al. described a DAPA-induced anti-atherosclerotic effect manifested by a lower inflammatory response and infiltration capacity of macrophages with attenuated progression of the plaque. Mechanistically, DAPA inhibited the Toll-like receptor 4 (TLR-4)/NF-κB signaling pathway along with IL-6 and TNF-α release [43].

Overall, DAPA emerges as a potent modulator of vascular inflammation and endothelial homeostasis by targeting interconnected pathways, including NF-κB/NLRP3 signaling, oxidative stress, AMPK/SIRT1 activation, NHE1 inhibition, and platelet reactivity. The consistency of these effects across normoglycemic and hyperglycemic conditions supports a glycemia-independent vasculoprotective role, potentially contributing to the cardiovascular benefits of SGLT2 inhibition in non-diabetic patients.

#### 2.1.2. Empagliflozin

EMPA is the most extensively studied SGLT2 inhibitor in the context of vascular inflammation and atherosclerosis, with a robust body of experimental evidence supporting its glycemia-independent effects. Across a wide spectrum of in vitro and in vivo models, EMPA has been shown to directly modulate endothelial cells, macrophages, vascular smooth muscle cells, and platelets, and target several processes, such as inflammation, oxidative stress, cellular senescence, thrombosis, and plaque stability.

In a study on RAW 264.7 macrophages challenged with LPS, Lee et al. described EMPA-associated anti-inflammatory effects manifested by suppression of M1 macrophage polarization, inhibition of cyclooxygenase-2 and the release of pro-inflammatory cytokines and chemokines, downregulation of inducible nitric oxide synthase (NOS) gene expression and blocked phosphorylation of NF-κB, JNK and STAT1/3. These anti-inflammatory properties were further enhanced by EMPA-gemigliptin co-treatment [44]. In a more recent work, Xu et al. reproduced and extended these beneficial results by revealing that EMPA induces autophagy in RAW246.7 macrophages, human aortic smooth muscle cells (HASMCs) and HUVECs by activating the AMPK signaling pathway. Consequently, autophagy delayed atherosclerosis progression by blocking HASMC proliferation and migration, inhibiting the foaming reaction in RAW246.7 cells and HASMCs and significantly decreasing the protein levels of TNF-α and IL-6 expressed by HUVECs and macrophages [45].

Exposure of porcine coronary artery endothelial cells to plasma collected from COVID-19 patients elicited the upregulation of the SGLT2 protein and a marked pro-oxidant response that was directly proportional to the high serum levels of IL-1β, IL-6, TNF-α and MCP-1, especially in the case of the acute disease [46]. In vitro EMPA treatment partially restored SGLT2 expression and potently mitigated the endothelial pro-oxidant response in long-term (24 h) cell exposure to COVID-19 plasma but proved ineffective in the case of short-term (30 min) experiments. This suggests that SGLT2 was most likely involved in maintaining, not initiating, the pro-oxidant signal. Moreover, EMPA blunted the stimulatory effect of COVID-19 plasma on the nuclear translocation of NF-κB and on the expression of genes associated with cell senescence, thrombosis and production of adhesion molecules and proinflammatory cytokines. Additionally, EMPA decreased COVID-19 plasma-induced thrombogenicity by improving the endothelial antiaggregatory effect and NO production together with the inhibition of endothelial platelet adhesion and thrombin generation [46]. The importance of this latter aspect was further substantiated by Hasan et al., who demonstrated that the exposure of porcine endothelial cells to thrombin upregulated SGLT2 protein expression and induced oxidative stress and endothelial senescence, which were all abrogated by EMPA [47].

The effects of EMPA on the inflammatory response triggered by persistent disruption of the endothelial glycocalyx were investigated in HAECs [48,49]. EMPA restored glycocalyx integrity after heparinase III treatment and inhibited the TNF-α-induced adhesion of neutrophil-like cells to HAECs in both cultured conditions as well as in steady wall shear stress experiments. A postulated mechanism of the glycocalyx-protective effect was the downregulation of unfolded protein response genes, a hallmark of the attenuated endoplasmic reticulum stress.

In a diet-induced obesity model, C57BL/6J mice were given EMPA at 3 mg/kg/day and 10 mg/kg/day for 16 weeks. In both liver and white adipose tissue, the therapy increased the expression of the anti-inflammatory M2 macrophage phenotype, reduced the growth of pro-inflammatory M1-polarized macrophages, and significantly decreased plasma TNF-α levels. These findings indicate that EMPA modulates macrophage polarization, contributing to decreased systemic inflammation and improved metabolic outcomes in obese mice [50].

Fu et al. investigated the diabetes-independent impact of EMPA on vascular atherosclerosis using both an in vitro model of oxidized low-density lipoprotein (ox-LDL)-induced macrophage inflammation and in vivo experiments on spontaneously atherosclerotic ApoE^−/−^ mice fed a high-fat diet. Post-therapeutically, decreased area of atherosclerotic plaques in the aortic tree, and lower levels of macrophage infiltration/foaming and local release of inflammatory mediators (NF-κB, IL-6, IL-1β) were reported. Moreover, the circulating concentrations of IL-6 and IL-1β were significantly attenuated, while treated macrophages exhibited improved AMPK phosphorylation and reduced mRNA expression of IL-1β, IL-6, TNF-α and MCP-1. The authors concluded that EMPA elicits anti-inflammatory and anti-atherosclerotic effects in a dose-dependent manner [51].

Han et al. assessed the effects of EMPA on the development of atherosclerosis in male ApoE^−/−^ mice fed a Western diet. At the 8 week treatment timepoint, apart from the improved blood glucose control, the authors noted that EMPA elicited a significant reduction in the area of aortic arch atherosclerotic plaques along with a corresponding decrease in their inflammatory cell infiltration. These salutary effects were paralleled by a decline in the circulating levels of TNF-α, IL-6, monocyte chemoattractant protein-1 (MCP-1) and serum amyloid A and by a lower concentration of urinary microalbumin [52]. Several other groups used the same animal model to confirm the EMPA-induced inhibition of atherosclerosis progression along with additional secondary endpoints. Thus, Liu et al. revealed that EMPA treatment modulates sympathetic activity and the renin-angiotensin-aldosterone system by decreasing the serum concentrations of renin, aldosterone and norepinephrine [53]. Dimitriadis et al. described an EMPA-associated reduction in VCAM-1 and MCP-1 mRNA expression in the aortic root, while at the atheroma plaque level, the collagenolytic matrix metalloproteinases (MMP) were partially outbalanced by their tissue inhibitors (TIMP), highlighting the putative role of EMPA in plaque stabilization [54]. A similar decrease in MMP-2 and -9 was described in an ApoE^−/−^ mouse model of abdominal aortic aneurysm where EMPA also mitigated NF-κB activation and macrophage infiltration at the aneurysm level. Supplementary in vitro experiments revealed attenuated leukocyte–endothelial cell interactions and release of inflammatory chemokines, respectively [55].

Atherosclerotic calcification is associated with a high risk of plaque rupture. In ApoE^−/−^ mice fed a Western diet and treated with EMPA for 24 weeks, atherosclerotic calcification was significantly attenuated via the inhibition of vascular smooth muscle cell (VSMC) osteogenic differentiation, re-emphasizing the role of SGLT2 inhibition in plaque stability [56].

Collectively, EMPA exerts broad anti-inflammatory and anti-atherosclerotic effects largely independent of glycemic control, by modulating macrophage polarization, suppressing NF-κB signaling, inducing AMPK-dependent autophagy, and limiting oxidative stress and thrombogenicity. In vivo ApoE^−/−^ models confirm reduced plaque burden, vascular remodeling, and calcification with enhanced plaque stability, supporting empagliflozin as a potent vasculoprotective agent beyond diabetes.

#### 2.1.3. Canagliflozin

CANA displays a distinct vascular and anti-inflammatory profile among SGLT2 inhibitors, partly attributable to its lower selectivity for SGLT2 over SGLT1. In experimental models of vascular inflammation and atherosclerosis, accumulating evidence suggests that CANA exerts potent anti-inflammatory, pro-autophagic, and plaque-stabilizing actions in both cellular and animal models, some of which involving mainly SGLT1-dependent mechanisms.

In NIH mice and LPS-challenged RAW264.7 and THP-1 cells, CANA elicited anti-inflammatory effects by inhibiting the production and release of IL-1, IL-6 and TNF-α and promoting autophagy via AMPK phosphorylation [57].

In human coronary artery endothelial cells (HCAECs), Uthman et al. demonstrated that CANA prevented the LPS-induced IL-6 release and ERK 1/2 phosphorylation via a decrease in hexokinase II expression. The CANA-mediated anti-inflammatory properties included the activation of AMPK, an effect which, surprisingly, was not present when HCAECs were treated with DAPA or EMPA [58]. These results were replicated by Mancini et al., who used an in vitro model of IL-1β-challenged HUVECs and HAECs to reveal that CANA, but not EMPA or DAPA, promoted AMPK phosphorylation, inhibited the secretion of IL-6 and MCP-1 and mitigated the adhesion of pro-monocytic U937 cells [59]. However, the EMPA and DAPA concentrations used by these groups were lower than those described in the papers citing a stimulating effect of these two SGLT2i on AMPK phosphorylation. Importantly, since CANA has the lowest selectivity for SGLT2 over SGLT1 out of all the commonly used SGLT2i [2], there is a strong possibility that its endothelial actions were mediated by the SGLT1 protein. Indeed, Kondo et al. have shown that CANA elicits AMPK phosphorylation in cardiomyocytes via SGLT1-related signaling [60].

In male ApoE^−/−^ mice fed a high-fat diet, CANA reduced both the area of aortic root atheroma plaques as well as the expression of MCP-1 and VCAM-1. The collagen content and the TIMP-1/MMP-2 ratio in the atherosclerotic lesions showed a higher post-therapeutic value, suggesting an improved plaque stability as a result of CANA treatment [61]. Using the same animal model, Zuo et al. described similar CANA-mediated benefits, namely smaller aortic root atherosclerotic plaque areas with higher collagen fiber content and less macrophage infiltration. Moreover, CANA promoted aortic autophagy and attenuated the serum levels of inflammatory cytokines (IL-1β, IL-6, TNF-α) [62].

Of note, in female ApoE^−/−^ mice fed a Western diet, CANA failed to affect the size of atherosclerotic plaques, indicating that hormonal factors might be responsible for this lack of effect and emphasizing the importance of addressing sex-related differences in experimental settings [63].

Similarly to EMPA, CANA seems to mitigate rat and human arterial ring calcification via decreased osteogenic differentiation of VSMC and suppression of the NLRP3 signaling pathway [64].

Collectively, CANA exhibits anti-inflammatory and anti-atherosclerotic effects through AMPK activation, autophagy induction, and suppression of pro-inflammatory signaling, which results in improved plaque stability and reduced vascular calcification in non-diabetic settings. Its lower selectivity for SGLT2 suggests partial involvement of SGLT1-dependent pathways, highlighting mechanistic heterogeneity and context-specific vascular effects within the SGLT2 inhibitor class.

### 2.2. Alleviation of Oxidative Stress and Mitochondrial Dysfunction

Oxidative stress and mitochondrial dysfunction are increasingly recognized as central drivers of endothelial injury, vascular inflammation, and atherosclerotic disease progression. Mitochondria are the main sources and targets of oxyradicals in both acute and chronic pathologies. Excessive mitochondrial ROS production disrupts redox homeostasis, impairs endothelial signaling, and amplifies pro-inflammatory cascades within the arterial wall. Emerging evidence suggests that mitochondrial protection represents a critical mechanism through which SGLT2i exert their vasculoprotective effects.

Apart from their crucial role in generating the ATP molecule through oxidative phosphorylation, mitochondria have long been described as key players in several signaling pathways influencing oxidative stress, inflammation, calcium handling and cell survival [65]. The organelles also play a pivotal role in vascular homeostasis since the cells of arterial walls are characterized by high metabolic activity; also, mitochondrial dysfunction is currently recognized as an important promoter of atherosclerosis [66]. Oxidative phosphorylation physiologically generates low amounts of ROS that serve as intracellular signaling molecules involved in the regulation of vascular function and endothelial integrity. ROS (superoxide anion, hydroxyl radical, H_2_O_2_) and reactive nitrogen species (RNS) are normally paired with endogenous antioxidant systems that neutralize them. Conditions leading to either increased ROS/RNS generation or inadequate activity of the antioxidant enzymes result in oxidative stress [67]. Consequently, a vicious cycle is created whereby mitochondrial dysfunction leads to low energy production and excessive release of ROS, which in turn, will damage the organelles, generating additional mitochondrial dysfunction and promoting local pro-inflammatory and pro-atherosclerotic conditions [66].

Given the positive clinical outcomes associated with SGLT2i treatment, there is great interest in describing the protection-affording mechanisms of this drug class, with a great body of work focusing on the impact of gliflozins on mitochondria. Numerous studies have outlined a strong potential of SGLT2i to alleviate mitochondrial dysfunction by reducing mitochondrial ROS generation [68], increasing mitochondrial biogenesis and mitophagy [69], and improving bioenergetics [70]. Specifically, SGLT2i mitigate mitochondria-derived oxidative stress by various mechanisms that include, but are not limited to, the reduction of the inhibitory effect of high glucose on Nrf2 signaling, decreased production of NADH by the Krebs cycle and reduced mitochondrial Ca^2+^ overload [71].

In conclusion, there is unequivocal preclinical evidence that SGLT2i directly protect mitochondrial function in vascular cells by reducing mitochondrial ROS, improving bioenergetics, and promoting mitophagy, thereby disrupting the vicious cycle of oxidative stress and vascular inflammation underlying atherosclerosis. These effects are largely independent of glucose lowering and are further detailed for DAPA, EMPA and CANA in non-diabetic experimental settings.

#### 2.2.1. Dapagliflozin

DAPA has emerged as a key SGLT2 inhibitor with pronounced mitochondria-targeting antioxidant effects in vascular cells. Beyond its anti-inflammatory actions, accumulating experimental evidence indicates that DAPA directly preserves mitochondrial integrity, improves mitochondrial dynamics and bioenergetics, and limits oxidative stress-induced endothelial injury. These mechanisms appear particularly relevant in conditions characterized by hypoxia, lipotoxicity, and pro-inflammatory stress, all of which are central to atherosclerosis progression in non-diabetic settings. At the endothelial level, these protective effects translate into preserved nitric oxide signaling and redox balance under inflammatory conditions. TNF-α reduces NO bioavailability and potentiates intracellular ROS generation in HCAECs. Uthman et al. showed that pre-incubation of these cells with EMPA or DAPA abolishes these negative effects in an SGLT2-independent manner that, instead of impacting endothelial NOS (eNOS) expression/signaling, barrier function, or adhesion molecule expression, restored NO bioavailability by preventing ROS production [72].

Hypoxia/reoxygenation (H/R) in HCAECs is associated with increased formation of fragmented mitochondria (enhanced mitochondrial fission and reduced fusion). Pre-treatment of endothelial cells with DAPA for 24 h prevented these detrimental effects and normalized the mitochondrial network [73].

Ferroptosis is a form of regulated cell death prompted by iron-dependent lipid peroxidation and currently recognized as an important contributor to endothelial dysfunction, initiation, progression and destabilization of the atherosclerotic plaques. In HUVECs exposed to oxidized LDL, DAPA stimulated the RAS-related protein 1B (RAP1B) signaling pathway, which subsequently alleviated ferroptosis, upregulated the NRF2, PGC-1α and mtTFA proteins, and elicited NRF2 nuclear translocation [74]. At the mitochondrial, level RAP1B pathway activation led to improved biogenesis, oxidative phosphorylation with enhanced ATP production, and decreased generation of mitochondrial ROS with oxidative stress alleviation. In ApoE^−/−^ mice, DAPA treatment reduced the size of the atherosclerotic plaques and the magnitude of vascular ferroptosis, and enhanced endothelial mitochondrial biogenesis and energy metabolism. Knocking out the RAP1B gene abolished these beneficial outcomes, highlighting the importance of this pathway for the DAPA-related protective effects [74].

In a recent elegant study, the group of Kutryb-Zajac reported that DAPA enhanced mitochondrial respiration and NO production in mouse cardiac endothelial cells (H5V) subjected to hypoxia-mimicking conditions. Interestingly, in vitro incubation with DAPA augmented only mitochondrial respiration, while glycolysis remained unchanged in endothelial cells. Moreover, they found that DAPA doubled the rate of coronary NO release and augmented coronary capillary density in female C57BL/6 mice [75].

He et al. investigated the effects of DAPA in obese nondiabetic mice fed a high-fat diet and on HUVECs, respectively. In vivo, DAPA significantly attenuated the obesity-induced endothelial dysfunction, while in palmitic acid-treated HUVECs, it prevented the decrease in mitochondrial membrane potential, viability and energetics and rescued mitochondrial biogenesis and structural integrity, possibly via activation of the SIRT1/PGC-1α signaling pathway [76].

Collectively, DAPA confers robust mitochondrial and antioxidant protection by preserving mitochondrial dynamics and biogenesis, limiting ROS generation and ferroptosis, and restoring NO bioavailability in endothelial cells and in vivo models. These pleiotropic, glucose-independent effects—partially mediated by RAP1B, NRF2, and SIRT1/PGC-1α signaling—have established DAPA as a potent modulator of mitochondrial health and vascular function in non-diabetic settings.

#### 2.2.2. Empagliflozin

Endothelial oxidative stress represents a common downstream pathway through which metabolic, inflammatory, mechanical, and ischemic insults converge to cause vascular function impairment. In this context, EMPA has been extensively investigated for its capacity to limit ROS generation and preserve endothelial viability under diverse stress conditions. Experimental studies have provided mechanistic insights into how EMPA modulates redox-sensitive signaling pathways, mitochondrial calcium handling, and endothelial barrier integrity in both static and dynamic vascular environments. These protective actions are well illustrated by experimental models directly linking oxidative stress to endothelial cell survival.

Oxidative stress undoubtedly impacts cellular viability. Mone et al. conducted a complex study that comprised two in vitro stages. Initially, they utilized HUVECs to prove that EMPA decreases high glucose-induced mitochondrial Ca^2+^ overload, ROS generation and endothelial leakage. Subsequently, they demonstrated that increased H_2_O_2_ concentrations reduce the viability of human brain microvascular endothelial cells and that pretreatment with EMPA mitigated this negative effect [68].

The endothelial monolayer is adapted to the normal mechanical forces created by the bloodstream, but in conditions of enhanced cyclic stretch, endothelial dysfunction follows. In an elegant study by Li et al., EMPA, DAPA and CANA mitigated the cell permeability increase, vascular endothelial cadherin degradation and ROS generation in HCAECs exposed to 10% stretch for 24 h. EMPA-related improvement of endothelial barrier function was due to decreased ROS release mediated, at least in part, via inhibition of NHE1 and the NADPH oxidases (NOXs) [77]. Extending these results, the authors unequivocally proved that the EMPA-triggered NOXs inhibition relies on attenuation of the NHE/Na^+^/NCX/Ca^2+^/Protein Kinase C axis [78]. The same Dutch group also showed that EMPA decreased TNF-α-induced ROS generation both in static cultured HUVECs and HAECs, as well as HCAECs under flow conditions via NHE1 inhibition with the subsequent decline in cytoplasmic sodium and calcium [79,80].

In cardiac microvascular endothelial cells (CMECs) subjected to ischemia/reperfusion (I/R) injury, EMPA treatment restored mitochondrial fission and fusion to normal levels, mitigated excessive mitochondrial ROS generation, attenuated mitochondrial permeability transition pore (mPTP) opening, stabilized mitochondrial membrane potential and suppressed mitochondrial apoptosis via activation of the AMPKα1/ULK1/FUNDC1 mitophagy pathway [81].

Although many studies have confirmed the mitochondria-protective effect of EMPA, there are also discrepant data in the literature. In aged (80-week-old) C57BL/6 J mice with established vascular dysfunction, Soares et al. demonstrated that a six-week course of EMPA significantly attenuated endothelial dysfunction and arterial stiffening with improved eNOS phosphorylation and reduced ROS generation. The authors found a potent downregulation of numerous pathways involved in ROS production and the metabolism of H_2_O_2_, along with a lower expression of the superoxide- and H_2_O_2_-producing enzyme xanthine oxidase. However, a shorter course of EMPA treatment did not impact the aging-induced aortic mitochondrial dysfunction, leading the authors to conclude that the attenuation of oxidative stress was mitochondria-independent [82].

Apart from the dysfunctional electron transport system, mitochondria contribute to cellular oxidative stress through enzymatic sources of ROS, such as the outer membrane monoamine oxidase (MAO) with two isoforms MAO A and B. In human internal mammary arteries harvested from non-diabetic overweight patients suffering from coronary heart disease, we have demonstrated that acute in vitro EMPA incubation reduced MAO A and B expression in both control vascular rings as well as in high glucose- and angiotensin II-challenged samples [83]. This resulted in a significant decrease in oxidative stress and improved endothelium-dependent relaxation of the arterial preparations. Of note, we have replicated these beneficial acute antioxidant effects in right atrial appendages harvested from overweight, non-diabetic cardiac patients and incubated with either EMPA or DAPA (concentrations relevant for the plasma level) [84], highlighting the potential of SGLT2i to mitigate oxidative stress at both the vascular and myocardial levels.

Overall, these findings indicate that EMPA exerts a multifaceted antioxidant effect in vascular cells, acting through both mitochondria-dependent and mitochondria-independent mechanisms. By limiting ROS generation under metabolic, inflammatory, mechanical, and ischemic stress, preserving endothelial barrier function, and modulating redox-related enzymatic sources such as NOXs, xanthine oxidase, and MAOs, EMPA effectively counteracts endothelial dysfunction.

#### 2.2.3. Canagliflozin

CANA has been shown to exert antioxidant and mitochondria-related effects in vascular cells, although its impact on mitochondrial function appears more heterogeneous when compared with other SGLT2i. Experimental studies have explored the ability of CANA to modulate oxidative stress, cellular senescence, and redox-sensitive signaling pathways in endothelial cells and atherosclerotic lesions, while also raising important concerns regarding its effects on cellular energy metabolism under certain conditions.

In an in vitro model of palmitic acid-induced vascular aging, CANA inhibited HUVEC cell cycle arrest, delayed cellular aging and mitigated ERK protein phosphorylation along with the associated ferroptosis by significantly attenuating the increment in intracellular ROS [85].

In male ApoE^−/−^ mice fed a high-fat diet, CANA treatment decreased ROS release from aortic root atherosclerotic plaques and modified the level of genes involved in oxidative stress. Thus, NADPH oxidase 4 mRNA expression was decreased, while the antioxidant NRF2 and glutathione S-transferase mRNA levels were significantly elevated. As a result, eNOS expression was upregulated, revealing the direct relation between oxidative stress attenuation and the improvement in endothelial function [62].

However, as opposed to other SGLT2i, in T2DM patients, CANA was associated with an increased risk of lower limb amputation [86]. Zügner et al. sought to explain the mechanisms behind this serious concern and found that especially at supra-pharmacological but also, to some extent, pharmacological concentrations, CANA inhibited HUVEC mitochondrial activity, reducing glycolysis, beta-oxidation and mitochondrial respiration and leading to an impaired cellular energy state that could be responsible for insufficient angiogenesis and therefore vascular complications. Importantly, DAPA and EMPA treatment did not display any of the above-mentioned detrimental effects [87].

Overall, CANA displays context-dependent effects on mitochondrial function and oxidative stress, attenuating ROS generation and endothelial senescence in atherosclerotic settings but potentially impairing endothelial bioenergetics at higher concentrations. This duality underscores the mechanistic heterogeneity of the SGLT2 inhibitor class and highlights the importance of dose and cell type when interpreting CANA’s effects, in line with the concept of personalized medicine.

### 2.3. Alleviation of Endothelial Dysfunction

Endothelial dysfunction represents a first pathophysiological event in the initiation of ATS and a central mechanism in the progression of most cardiovascular diseases. Under physiological conditions, the endothelium functions as a gatekeeper of vascular homeostasis by primarily regulating vascular permeability and vasomotor tone, along with vascular smooth muscle cell (VSMC) growth and the control of hemostasis [16]. Disruption of these tightly controlled processes leads to endothelial dysfunction, a pathological state characterized by reduced nitric oxide availability and impaired vasodilation, aberrant VSMC proliferation and migration with subsequent neointima formation, excessive oxidative stress and inflammation, platelet activation and a pro-coagulant status, and, in the long run, endothelial senescence. Viewing the essential role of the endothelium in cardio-metabolic diseases, pharmacological strategies with pleiotropic effects are of major therapeutic interest. As such, SGLT2i favorably impact survival and proliferation of the endothelial cells beyond metabolic control, positioning this class of drugs as crucial modulators of vascular health in both diabetic and non-diabetic settings [24,88].

Numerous preclinical studies have outlined the capacity of SGLT2i to restore the beneficial properties of the injured endothelium. For instance, in the presence of pro-atherogenic stimuli such as 25-hydroxycholesterol, EMPA, DAPA and CANA were all effective in maintaining HUVEC cell integrity [89]. Moreover, as detailed below, independently of glucose control and possibly even of SGLT2 activation, gliflozins enhance endothelial viability and barrier function, eNOS expression/activity, and NO bioavailability with improved vasodilation via endothelial-dependent or -independent mechanisms. Additionally, SGLT2i suppress oxidative stress and pro-inflammatory pathway activation, downregulate the expression of endothelial adhesion molecules, and inhibit VSMC proliferation, migration and neointima formation. A number of clinical trials also support the hypothesis that SGLT2i provide cardiovascular protection, at least partially via mitigating endothelial and microvascular dysfunction [20]. Moreover, the SGLT2i-induced attenuation of inflammation and endothelial dysfunction blunts vascular leakage, an effect which synergizes with the drug-induced natriuresis and diuresis to alleviate edema formation, thus improving cardiac function and patient quality of life [90,91].

The endothelial effects of DAPA, EMPA, and CANA reported in the literature are further summarized, highlighting both shared pathways and compound-specific mechanisms that contribute to their vasculoprotective actions in non-diabetic experimental settings.

#### 2.3.1. Dapagliflozin

In human aortic endothelial cells challenged with TNF-α, DAPA treatment stimulated the pro-survival PI3K/AKT pathway, eliciting an improvement in cellular resistance and promoting repair mechanisms. Moreover, in this inflammatory environment, DAPA restored eNOS expression levels and enhanced NO bioavailability, thus diminishing the TNF-α-induced endothelial dysfunction [38]. Similar results were reported in H_2_O_2_-challenged HUVECs where DAPA decreased intracellular ROS and peroxynitrite accumulation, improved eNOS activity and NO bioavailability and delayed cellular senescence via SIRT1 activation. Separately, in organ bath studies on aortic rings obtained from C57BL/6 J mice, acute DAPA treatment elicited vasorelaxation, supporting its direct role in the attenuation of endothelial dysfunction [92].

Hypertensive Dahl salt-sensitive rats fed a high-salt diet displayed endothelial overexpression of the adhesion molecules VCAM-1 and E-selectin and downregulation of eNOS as markers of endothelial dysfunction [93]. DAPA treatment for 6 weeks elicited a partial normalization of these expression profiles. Moreover, DAPA decreased the level of NHE1 present in whole heart lysates. To establish the cells primarily involved in this result, the authors conducted a supplementary line of experiments on HUVECs and demonstrated that DAPA significantly impacts NHE1 activity in endothelial cells.

In mice undergoing cardiac I/R injury, Ma et al. described microvascular ultrastructural changes and swollen endothelial cells with DNA fragmentation and overexpression of ICAM-1. DAPA administered for 7 days prior to I/R injury induction mitigated these detrimental effects. Moreover, extending their study to HCAECs subjected to H/R, this group demonstrated that 24 h of DAPA pre-treatment supported cell viability and counteracted apoptosis, restored eNOS activity and VEGF levels and attenuated endothelin-1 overexpression and cellular hyperpermeability. By preventing the xanthine-oxidase-induced SERCA2 inactivation and subsequent normalization of the intracellular calcium balance, DAPA regulated the CaMKII/cofilin pathway and preserved cytoskeletal integrity [73].

Gaspari et al. found that endothelium-dependent vasorelaxation of the abdominal aorta was improved by chronic DAPA treatment (4 weeks) in male ApoE^−/−^ mice fed a high-fat diet. Additionally, DAPA mitigated NF-κB activation and reduced ICAM-1 expression and macrophage infiltration at the aortic wall level, revealing anti-inflammatory effects associated with the attenuated endothelial cell dysfunction [34]. However, in this experimental model, as opposed to literature data, the atherosclerotic plaque area remained unchanged post-therapeutically. This might be partially due to the shorter treatment period applied by the authors.

In abdominal aortic rings isolated from C57Bl/6J mice fed a normal fat diet the same group revealed that acute DAPA treatment prompted endothelium-independent vasorelaxation [34]. This result is in agreement with several other studies. Li et al. postulated that DAPA promoted a dose-dependent vasodilator effect in male New Zealand rabbit thoracic aortic rings via the activation of the smooth muscle cell voltage-gated potassium Kv1.5 channel and protein kinase G [94]. The authors ruled out the involvement of the endothelium, other potassium channels, calcium channels, intracellular calcium or protein kinase A in the DAPA-induced vasodilation.

Similarly, in male Sprague Dawley rats, DAPA elicited concentration-dependent relaxation in small mesenteric arteries via the direct activation of the Kv7 potassium channel isoform, irrespective of the presence of intact endothelium or the inhibition of the SGLT2 protein [95]. In left descending coronary artery rings isolated from male Sprague Dawley rats, DAPA induced dose-dependent vasodilation regardless of the endothelium. Interestingly, at variance from the previous studies, potassium channel blockers did not alter the vasomotor response. Instead, DAPA inhibited extracellular Ca^2+^ influx and therefore decreased the phosphorylation of the 20 kDa myosin light chain, an important participant in smooth muscle contraction [96].

In neurogenic hypertensive Schlager (BPH/2J) mice treated with DAPA for 2 weeks, endothelium-dependent relaxation was improved via the decreased level of asymmetric dimethylarginine, the endogenous inhibitor of NOS [97].

Importantly, the acute protective effects of DAPA have been recently demonstrated in a murine model of postischemic recovery after cold cardioplegic arrest. It is well known that cold ischemia-reperfusion markedly impairs both systolic and diastolic function. Pre-ischemic administration of DAPA significantly enhanced left ventricular functional recovery, as evidenced by improved contractility, increased aortic flow, and better diastolic performance. In contrast, adding DAPA solely to the cardioplegic solution did not produce comparable benefits. High-energy phosphate (HEP) analysis revealed a significant increase in myocardial energy charge when DAPA was delivered through cardioplegia. These functional and metabolic improvements were further supported by enhanced cell viability and elevated HEP levels in HUVECs (and cardiac myoblasts) pre-treated with the SGLT2i [98].

#### 2.3.2. Empagliflozin

In an in vitro model of co-culture of human CMECs with adult rat cardiomyocytes, Juni et al. demonstrated that CMEC-derived NO positively regulates the ability of adult rat cardiomyocytes to contract and relax. This beneficial effect was abrogated by the pre-incubation of CMECs with TNF-α or IL-1β and restored by the addition of EMPA, which improved NO bioavailability not by influencing the eNOS protein content but by reducing mitochondrial ROS generation and, subsequently, the interaction between cytoplasmic ROS and NO [99]. In a later study, Juni et al. used the same experimental model to prove that uremic serum obtained from chronic kidney disease patients mitigated the endothelial enhancement of cardiomyocyte function. Pre-treatment of CMECs with EMPA inhibited the uremic serum-induced increment in mitochondrial ROS generation and decrease in NO bioavailability, respectively [100], suggesting that non-diabetic patients with chronic kidney disease might derive cardiovascular benefits from SGT2i therapy.

In human microvascular endothelial cells incubated with EMPA for 24 h and then exposed to H/R, the post-therapeutic cell survival was significantly increased via a STAT-3-mediated mechanism. In vivo experiments on non-diabetic C57BL/6 mice treated for 6 weeks with EMPA before being subjected to I/R confirmed this beneficial effect. Moreover, independently of STAT-3 activation, EMPA also reduced endothelial oxidative stress [101].

The occurrence of the ALDH2*2 allele of the ALDH2 enzyme is the most frequent enzymopathy caused by a single nucleotide polymorphism. It is also associated with a very high risk of coronary artery disease (CAD) and ATS. In iPSC-derived endothelial cells (iPSC-ECs) isolated from human subjects carrying the ALDH2*2 allele, Guo et al. described a reduced ALDH2 enzymatic activity with accumulation of the toxic aldehyde 4-hydroxynonenal, increased cellular ROS and monocyte-endothelial adhesion along with decreased NO bioavailability and impaired tube formation. In a second set of experiments, the same group revealed that aortic rings isolated from ALDH2*2 knock-in mice showed a reduced endothelial-dependent relaxation. However, EMPA mitigated ALDH2*2-associated endothelial dysfunction via inhibition of NHE1 and activation of the AKT/eNOS pathway [102].

The group of Daniel Sedding performed an elegant study that addressed the effects of EMPA on vascular function in human cells and on vascular remodeling in a murine model of vascular injuries [4]. Non-diabetic human coronary artery VSMCs, but not the endothelial cells (EC), incubated with EMPA for 24 h displayed a dose-dependent reduction in their migration and proliferation capacity with no evidence of apoptosis. In line with these observations, in vivo experiments in non-diabetic C57BL/6 mice revealed that pre-treatment with EMPA impaired neointima formation after wire-induced femoral artery injury but did not promote re-endothelialization after electric de-endothelialization of the left carotid artery. However, both of these beneficial effects were recorded in the case of diabetic animals, suggesting that even though SGLT2i are particularly protective in the presence of diabetes, their administration in its absence is also vasculoprotective.

Chang et al. recently published concordant results in adult male nondiabetic Wistar rats treated with EMPA 4 days prior and 14 days after the induction of carotid artery balloon injury. Post-therapeutically, balloon injury-induced neointima formation was significantly attenuated. In cultured VSMCs, EMPA decreased PDGF-mediated cellular proliferation and migration without eliciting apoptosis or cytotoxic death. Importantly, these authors demonstrated that the vasculoprotective effects of EMPA were independent of the SGLT2 inhibition or glycemic control [103].

Using male rabbit thoracic aorta rings, Seo et al. revealed that acute EMPA treatment evoked a dose-dependent vasodilating response regardless of the presence of the endothelium. This effect was mediated by the protein kinase G (PKG) signaling pathway and by the activation of the voltage-dependent potassium channel Kv, but not of the ATP-sensitive K^+^ (K_ATP_) channels, the large-conductance Ca^2+^-activated K^+^ (BK_Ca_) channels or the inwardly rectifying K^+^ (Kir) channels [104]. These results are in agreement with a later study published by Hasan et al., who used resistance-size rat mesenteric arteries to prove that acute EMPA administration elicits endothelium-independent vascular relaxation via Kv1.5 and Kv7 potassium channel isoforms, with no involvement of BK_Ca_ or K_ATP_ channels [105].

In a mouse experimental model of left ventricular pressure overload induced by transverse aortic constriction, EMPA administration mitigated the catecholamine-induced endothelial cell apoptosis and maintained capillarization in the heart after injury via activation of the AKT/eNOS/NO pathway in endothelial cells [106]. A subsequent study by Bruckert et al. performed on a murine model of angiotensin II-induced hypertension demonstrated that EMPA improved the eNOS/ROS balance at both the aortic macrovascular as well as mesenteric microvascular levels [107]. Moreover, EMPA mitigated the ACE/AT1R/NADPH oxidase pathway activation, decreased the endothelial expression of VCAM-1 and MCP-1 and displayed anti-remodeling properties by attenuating collagen I formation and downregulating MMP-2 and -9 levels at both vascular study sites. Interestingly, EMPA reduced the angiotensin II-increased endothelial expression of SGLT1 and SGLT2 both in the aorta as well as in the mesenteric and coronary vessels.

In a comprehensive study by Kolijn at al. carried out on samples harvested from male patients diagnosed with heart failure (HF) with preserved ejection fraction (HFpEF), EMPA decreased myocardial inflammation and oxidative stress, resulting in improved NO bioavailability, activity of the NO-sGC-cGMP cascade, and PKG activity (by decreasing PKGα oxidation). In a set of experiments performed on vascular samples isolated from these patients, EMPA evoked endothelium-dependent vasodilation without affecting smooth muscle vasorelaxation [108].

#### 2.3.3. Canagliflozin

In rat resistance-size mesenteric arteries, Hasan et al. reported the CANA-evoked endothelium-independent vasodilation, which was associated with a decrease in systolic and diastolic blood pressure after in vivo single-dose drug administration. This vascular tone modulation was not reliant on endothelial NO-sGC-PKG and prostacyclin signaling, SERCA pump stimulation or SGLT2 inhibition, but it instead depended on activation of smooth muscle potassium channels Kv1.5, Kv7 and Kv2.1 [109]. However, Sayour et al. reported that preincubation of non-diabetic rat thoracic aorta segments with CANA prior to contraction with phenylephrine resulted in endothelium-dependent relaxation. In a separate line of experiments, the same authors induced myocardial I/R injury in non-diabetic rats via left anterior descending coronary artery ligation and revealed that CANA administration after 5 min of ischemia significantly improved eNOS phosphorylation in left ventricular samples from the area at risk [110].

In a model of vascular I/R injury in non-diabetic male Wistar rats, Korkmaz-Icöz et al. demonstrated that CANA treatment improved endothelium-dependent vasorelaxation and displayed anti-inflammatory and anti-oxidant properties. Mechanistically, CANA decreased the expression of ICAM-1, downregulated the IL1a, IL6 and NADPH oxidase organizer 1 genes, prevented the I/R-induced upregulation of CD40 and increased the expression of the PECAM-1 endothelial marker. Given these positive outcomes, the authors hypothesized that CANA might have beneficial applications in patients undergoing coronary artery bypass grafting, where I/R injury is the leading contributor to tissue damage [111].

In a complex study, Han et al. highlighted the dependency of CANA treatment outcome on the type of vascular bed, the duration of the treatment and the presence of comorbidities such as diabetes. Acute incubation of non-diabetic mouse pulmonary artery rings with CANA attenuated sodium nitroprusside-induced membrane hyperpolarization and potassium channel activation in VSMC, and consequently inhibited endothelial-independent vasodilation; no effect was reported on the small coronary artery vasomotor capacity. At variance, in T2DM mice, chronic treatment with CANA for 4 weeks significantly improved coronary endothelial-independent vasodilation but displayed no benefits regarding vascular relaxation capacity in pulmonary arteries [112].

As previously stated, clinical studies have linked CANA to a risk of lower limb amputation. In preclinical conditions, although CANA displayed anti-inflammatory properties in HUVECs via the increase in heme oxygenase-1 expression and activity, this drug also elicited a concentration-dependent inhibition of HUVEC proliferation and migration [113]. In the absence of these processes essential for angiogenesis, limb blood flow might be minimized, explaining the amputation risk in the diabetic population. Similarly, Behnammanesh et al. reported that clinically relevant concentrations of CANA impaired DNA synthesis and blocked the cell cycle in the G_0_/G_1_ phase in HUVECs, partially by reducing the expression of cyclin A [114]. This resulted in an anti-proliferative effect that was further confirmed in HAECs and mouse aortic endothelial cells alike. Moreover, CANA marginally inhibited endothelial cell migration at high doses but potently reduced HUVEC tube formation and blocked the sprouting of EC capillaries, suggesting diminished neoangiogenesis. Endothelial tube formation was not affected by EMPA and DAPA.

In rat and human aortic VSMCs, clinically relevant concentrations of CANA mitigated cell proliferation and migration in a cytostatic rather that cytotoxic manner, as the authors noticed reduced DNA synthesis and arrest of VSMC development in the G_0_/G_1_ phase [115]. The anti-proliferative and anti-migratory properties of CANA were dependent on the activation of the ROS/Nrf2 pathway that stimulated the expression of heme oxygenase-1. As opposed to other studies, in this experimental model, EMPA and DAPA had no effect on the investigated parameters. However, it is important to mention that this study was conducted under non-stressful conditions, and the results might differ in the presence of injurious factors that stimulate the cells and activate different pro-inflammatory or pro-oxidant pathways.

Last but not least, De Stefano et al. performed an elegant study in visceral adipose tissue arteries obtained from obese and non-obese individuals and reported that CANA elicited a dose-dependent and endothelium-independent vasodilating effect (greater than the one elicited by the GLP1 receptor agonist liraglutide), which was slightly more potent in the obese population and, possibly, mediated by the inhibition of NHE1 in VSMCs [116].

The “off-target” vasculoprotective effects of the main SGLT2i (DAPA, EMPA and CANA) are summarized in Table 1.

## 3. Glycemia-Independent Vasculoprotective Mechanisms of Dual SGLT1/2 Inhibitors

In contrast to the substantial body of work focusing on SGLT2, SGLT1 has enjoyed minimal attention, especially concerning the effects of its modulation on blood vessel structure and function [118]. SGLT2 is responsible for 90% of glucose reabsorption in the kidney, while SGLT1 handles the remaining 10% load along with intestinal glucose reabsorption [2]. Accordingly, targeting hyperglycemia by blocking SGLT-dependent mechanisms seemed a promising new approach in the search for more effective antidiabetic treatments. However, when renal SGLT2 is inhibited, the reabsorption of the higher glucose load delivered to the distal part of the proximal tubule is partially compensated by SGLT1 [3], causing a urinary glucose excretion of only ~60% [119] out of the theoretically expected ~90% magnitude. Thus, it was hypothesized that dual SGLT1/2 inhibition would elicit greater glucosuria and further improve glycemic control by simultaneously targeting the kidney as well as the intestine. Moreover, in conditions of health, SGLT1 has a much wider tissue distribution that includes the central nervous system [2], the myocardium [120], the vessel wall [121], platelets and lymphocytes [122]. Cardiac disorders such as myocardial ischemia, hypertrophy [120], and dilated cardiomyopathy [123] and vascular pathologies including aging, inflammation, oxidative stress and hypoxia [124,125] upregulate SGLT1 expression, which further exacerbates endothelial dysfunction, vascular remodeling and inflammation [118,120]. Additionally, although complete loss of function of intestinal SGLT1 is fatal, genetic missense variants that induce partial functional impairment are associated with a lower risk of DM and HF occurrence and consequently with a reduced mortality rate [123]. Therefore, dual SGLT1/2 inhibition can provide additional benefits to the already established significant improvement in glycemic parameters and cardiovascular outcomes afforded by single SGLT2i therapy [126].

### 3.1. Sotagliflozin (SOTA)

Although SGLT2 inhibitors display a strong affinity for this transporter, they also simultaneously target SGLT1 to a different extent [126]. As such, the selectivity profile for SGLT2 over SGLT1 ranges from 250-fold for CANA, the first SGLT2i to be successfully marketed in Europe and the U.S. [127], to 2700-fold for EMPA at the opposite end of the spectrum [126]. At variance, SOTA registers a mere 20-fold selectivity for SGLT2 over SGLT1 [126] and is nowadays recognized as the first dual SGLT1/2i to show clear cardiovascular benefits apart from its efficacy in diabetes management [128]. Moreover, given the wide tissue distribution of SGLT1, the dual inhibition approach might be more beneficial in terms of afforded nephro- and neuroprotection [129]. Despite the relatively limited experience with its use, SOTA is reported to possess a safety profile comparable to other SGLT2i [130], and emerging studies have provided promising results regarding its beneficial effects on vascular function, contributing to reduced atherothrombotic risk, as demonstrated by outcome trials [128].

Khemais-Benkhiat et al. revealed that SOTA (but not EMPA) significantly reduced basal glucose uptake into control porcine coronary artery endothelial cells, indicating a major role for SGLT1 in physiological conditions [125]. Moreover, exposure of the endothelial cells to H_2_O_2_ resulted in enhanced glucose entry and upregulation of SGLT1 and 2 protein expression. Both EMPA and SOTA significantly decreased the ROS-stimulated glucose uptake, hence inhibiting endothelial glucotoxicity [125].

The complex inter-relation between angiotensin II (Ang II) and SGLT1 and 2 isoforms has been evaluated by several studies.

Sherratt et al. reported that under normal glucose conditions, a 24 h treatment of HUVECs with Ang II increased the expression of both SGLT1 and SGLT2, as well as of proteins related to inflammation and NADPH-oxidase mediated oxidative stress [131]. Pretreatment with SOTA 30 min prior to the Ang II challenge mitigated these detrimental effects by reducing the expression of SGLT2 along with that of the pro-oxidant p22phox, eNOS-inhibiting caveolin-1, and of proteins linked to the pro-inflammatory Akt pathway. Moreover, in a previous study, the same group compared the ROS detoxification capacity of SOTA and EMPA after challenging HUVECs with either IL-6 or LPS [132]. The authors revealed that EMPA modulated the expression of significantly fewer proteins involved in protection against oxidative stress, with no effect on the antioxidant enzymes peroxiredoxin, glutathione peroxidase-1 or thioredoxin. Conversely, SOTA had an important yet dichotomic effect on these specific proteins depending on the inflammatory challenge. As such, in response to IL-6, SOTA upregulated the level of peroxiredoxin and glutathione peroxidase-1, while in response to LPS, SOTA decreased their expression but stimulated that of thioredoxin, underscoring the ability of dual SGLT1/2 inhibition to diminish vascular oxidative stress during inflammation [132].

Park et al. reported that Ang II dose-dependently upregulated the expression of SGLT1 and 2 in endothelial cells isolated from porcine coronary artery and rat arterial segments and caused oxidative stress that was inhibited by SOTA, EMPA, NADPH oxidase inhibition, and AT1 receptor antagonism (losartan), respectively [121]. In the absence of extracellular glucose and Na^+^, the long-term Ang II-induced pro-oxidant response was blocked, suggesting that SGLT1 and 2 contribute to enhance their own expression. While the two SGLT2i mitigated the up-regulation of SGLT1 and 2 as well as the endothelial and arterial oxidative stress elicited by long-term cell exposure to Ang II, they were ineffective in the case of short-term (30 min) endothelial challenge experiments, indicating that SGLT1 and 2 were most likely involved in perpetuating and not initiating the sustained ROS formation. Moreover, SOTA and EMPA inhibited the ACE/AT1R/NADPH oxidase pathway activation and blunted the pro-senescence response, the downregulation of eNOS and NO formation and the upregulation of VCAM-1, MCP-1 and tissue factor that were induced by Ang II treatment. In a separate set of experiments, these authors reported that blood-derived microparticles collected from patients with CAD enhanced the endothelial expression of SGLT1, SGLT2 and VCAM-1 and downregulated eNOS protein level and NO formation, effects that were reversed by both SOTA and EMPA treatment [121].

Bruckert et al. also confirmed the vascular pro-oxidant effect of Ang II and demonstrated its role as a potent inducer of endothelial SGLT1 and 2 expressions at the level of different vascular sites [107]. Accordingly, high levels of both isoforms were registered in the rat macro-vasculature, namely the thoracic aorta, but only SGLT1 was overexpressed in murine mesenteric and coronary micro-vessels. Moreover, vascular oxidative stress was reduced by SOTA treatment and abolished by EMPA administration. The latter SGLT2i also downregulated the expression of SGLT1 and 2. However, as evaluation of the protective role of EMPA represented the main focus of this study, the authors did not investigate the effect of SOTA on the endothelial SGLT1 and 2 protein levels [107].

Recently, Lyu et al. reported that SOTA blocked the pro-oxidative response, endothelial NO production impairment, nuclear NF-κB translocation, and enhanced adhesion capacity of THP-1 monocytes and platelets in porcine coronary artery endothelial cells challenged with LPS [133].

In an elegant study, the group of Schini-Kerth et al. confirmed the link between SGLT1 and 2 expression and low-grade inflammation in human vasculature. In internal thoracic artery segments harvested from patients diagnosed with CAD, the authors reported that SGLT1 and 2 expressions were increased, both at the level of intima as well as in the media, and they were positively correlated with the levels of IL-1β, IL-6, TNF-α and p-p65 NF-Κb [134]. The vascular areas with the highest SGLT2 expression displayed significant ROS generation, which was mitigated by both SOTA and EMPA. Similar results were obtained in a separate line of experiments involving porcine coronary artery endothelial cells where exposure to IL-1β, IL-6 and TNF-α upregulated the expression of SGLT1 and 2, VCAM-1, ACE1 and AT1R and downregulated eNOS protein levels, respectively. Moreover, TNF-α enhanced endothelial glucose uptake and NF-κB activation. SOTA and EMPA prevented these detrimental effects and preserved NO formation in response to bradykinin. Interestingly, the knockdown of SGLT2 decreased the expression of SGLT1 and VCAM-1 and increased that of eNOS. Conversely, neither the latter two parameters nor the SGLT2 protein level were influenced by SGLT1 knockdown. AT1R inhibition in TNF-α-challenged cells blocked the pro-oxidant pathway and the upregulation of SGLT1 and 2, proving that oxidative stress is necessary to promote SGLT overexpression [134].

Endoplasmic reticulum stress is a major determinant of endothelial dysfunction via the activation of pro-oxidant and pro-inflammatory pathways. In HAAECS challenged with tunicamycin, Campeau and Leask reported that pre-incubation with SOTA completely abolished (while EMPA only attenuated) the expression of endoplasmic reticulum stress markers thioredoxin interacting protein (TXNIP) and NLRP3, which have been associated with oxidative stress and inflammation [135].

Aging was linked to endothelial SGLT1 upregulation at the level of the rat aortic arch and aorta, which promoted vascular oxidative stress. Both SOTA and an anthocyanin-rich blackcurrant concentrate limited the pro-oxidant response and the increase in systolic blood pressure. Interestingly, SOTA inhibited the aortic uptake of anthocyanins, which led the authors to conclude that the intracellular transport of these phytochemicals depends on SGLT1 [136].

In the clinical arena, although SGLT2 and SGLT1/2 inhibitors both induce cardiovascular benefits and share the ability to decrease the risk of major adverse cardiovascular events in diabetic patients, SOTA was proven to additionally reduce the incidence of atherothrombotic events, such as myocardial infarction and stroke [137]. Various mechanisms might be responsible for this ischemic benefit. SOTA dose-dependently inhibits platelet activation, aggregation, adhesion and thrombus formation without influencing coagulation parameters or bleeding potential [138]. Hasan et al. demonstrated that the exposure of porcine atrial tissue endothelial cells to thrombin upregulated the low protein expression level of SGLT1 and induced oxidative stress and endothelial senescence, which were abrogated by SOTA [47]. Moreover, Stanger et al. recently compared the antithrombotic effect of SOTA with that of the SGLT2-selective EMPA. Neither inhibitor altered the level of coagulation parameters or the bleeding time. EMPA was minimally effective in mitigating platelet aggregation in vitro and post-injury intravascular thrombus formation in vivo, while SOTA significantly impacted these parameters with the added benefit of in vitro inhibition of platelet activation, adhesion and thrombus formation. This discrepancy in the drug-related antithrombotic profile underscores a major role for the SGLT1 transporter [139]. Additionally, unlike SGLT2i, SGLT1 inhibition potentiates glucose delivery to the distal intestine, which modulates the local pH and consequently alters the microbiome. This leads to accumulation of short chain fatty acids that stimulate the secretion of glucagon-like peptide-1 (GLP-1) [140]. GLP-1 receptor agonists are reported to improve endothelial function, reduce inflammation, enhance atherosclerotic plaque stability [141] and attenuate platelet aggregation [142]. Thus, enhanced GLP-1 availability is likely an important contributor to the antithrombotic protection afforded by SGLT1 inhibition. Moreover, SGLT2 inhibitor treatment stimulates renal and hepatic erythropoietin synthesis and thus increases the hematocrit and blood viscosity [143], which can partially explain the low to absent effect of this drug class on the incidence of myocardial infarction and stroke [126].

### 3.2. Canagliflozin, Phlorizin and Mizagliflozin

Canagliflozin is the SGLT2i with the greatest impact on SGLT1. A recent study performed by Chen et al. revealed that CANA was able to mitigate monocrotaline-induced pulmonary artery hypertension in rats by interacting with SGLT1 [124]. Specifically, CANA treatment reduced pulmonary artery structural remodeling, as evidenced by the improvement in echocardiographic parameters and the diminished thickness of the vascular wall as a result of the significant reduction in inflammatory cell infiltration and muscle cell activity. At the molecular level, these authors reported that untreated pulmonary artery segments presented abundant expression of SGLT1, while SGLT2 was nearly undetectable. Monocrotaline upregulated the level of SGLT1 and Proliferating Cell Nuclear Antigen (PCNA), a marker of cell proliferation, and elicited a decrease in AMPK activation. CANA reversed these detrimental effects and thus mitigated pulmonary artery remodeling via the modulation of the SGLT1/AMPK signaling pathway. Similarly, in rat and human pulmonary artery smooth muscle cells (PASMCs) challenged with PDGF-BB or subjected to hypoxia, these authors also found that CANA exerted an anti-proliferative effect by downregulating PCNA expression and enhancing AMPK activation in a time- and concentration-dependent manner. SGLT1 knockdown interfered with the protective mechanisms elicited by CANA, leading the authors to conclude that the drug-related anti-remodeling effects in the pulmonary artery are dependent on the regulation of the SGLT1/AMPK signaling pathway [124].

Phlorizin is a dual SGLT1/2 inhibitor that displays a 6-fold selectivity for SGLT2 over SGLT1. Although very effective in blood glucose management, its development as an oral treatment for diabetes was limited by its poor solubility and low bioavailability [144]. However, several studies have exploited its dual inhibitory potential to investigate the involvement of SGLT1 and 2 in vascular pathology.

More than two decades ago, in HUVECs subjected to hypoxia, Berna et al. studied the mechanistic sequence connecting the decrease in ATP to the increase in cytosolic Ca^2+^ concentration, which subsequently activates phospholipase A2 and the release of prostaglandins and platelet-activating factor [145]. The authors elegantly showed that the reduced ATP and energetic availability was compensated by glycolysis activation with glucose uptake via SGLT, leading to intracellular Na^+^ accumulation. The consequent activation of the Na^+^/Ca^2+^ exchanger finally resulted in an increased concentration of cytosolic Ca^2+^. Phlorizin decreased the intracellular Ca^2+^ level as well as the activation of phospholipase A2, demonstrating an important role for SGLT1 and 2 in hypoxia-induced endothelial damage [145].

Ishida et al. used the asymmetric common carotid artery surgery to induce vascular cognitive impairment in mice and proved that although phlorizin and the SGLT1-selective inhibitor mizagliflozin both ameliorated cognitive function, only phlorizin was able to improve cerebral blood flow after surgery and decrease the expression of SGLT1, IL-1β and TNF-α in the brain [146]. Intense glucose uptake into ischemic cells has been demonstrated to be detrimental due to the occurrence of acidosis and ROS overproduction. In confluent bovine brain microvascular endothelial cells cocultured with astrocytes, Vemula et al. showed that oxygen and glucose deprivation elicited an increase in SGLT1 immunoreactivity and glucose intracellular transport [147]. Phlorizin significantly reduced glucose uptake both in this in vitro experimental line as well as well as in an in vivo mouse middle cerebral artery occlusion model where phlorizin proved effective in mitigating glucose blood-to-brain transport and reducing brain infarct and edema areas in the context of focal ischemia [147]. These results outline a possible role for SGLT inhibition in stroke.

In order to improve the therapeutic efficacy and bioavailability of phlorizin, Wu et al. recently developed a novel drug delivery system under the form of phlorizin liposomes and demonstrated that this formulation possessed exceptional physical structure, slow release and biocompatibility [148]. These authors reported that phlorizin liposomes significantly reduced vascular inflammatory and foam cell infiltration and improved the endothelial structure in a rat model of carotid atherosclerosis. Moreover, this formulation elicited a decrease both in the serum level of total, LDL and HDL cholesterol and triglycerides as well as in vascular lipid deposition. Phlorizin-liposomes attenuated the inflammatory response by downregulating the expression of phosphorylated NF-κB, IL-1β, TNF-α, COX-2 and iNOS at the level of the carotid atherosclerotic plaques. Additionally, the treatment reduced the carotid cellular damage induced by oxidative stress by augmenting the expression of Nrf2 and of its downstream antioxidant enzymes NAD(P)H quinone oxidoreductase 1, heme oxygenase 1 and glutamate-cysteine ligase catalytic subunit (GCLC) [148].

CD4+ T cells and interferon-γ are important participants in the pro-inflammatory vascular response and play a significant role in atherosclerotic plaque progression and destabilization [149]. Recently, Jin et al. reported that phlorizin and EMPA reduced glucose uptake into activated human CD4+ T cells and that phlorizin was approximately two times more efficient in this regard than EMPA at both normal and high glucose concentrations in the pericellular milieu. Moreover, phlorizin blunted the release of interferon-γ from CD4+ T cells irrespective of the presence of insulin and glucose conditions, while EMPA was only effective in high-glucose experiments, demonstrating yet again an important anti-inflammatory effect associated with SGLT inhibition [150].

Vascular tone and function are contingent not only on endothelial and smooth muscle cell behavior but also on the perivascular components. Preadipocytes, the main cellular component of perivascular adipose tissue, are capable of releasing various pro-inflammatory cytokines involved in atherosclerosis and vascular remodeling. Liu et al. used lentiviral vectors to silence or overexpress the SGLT1 gene in these cells and study their effect on mouse carotid arteries. The authors demonstrated that SGLT1 upregulation inhibited apoptosis and augmented cell glucose uptake, proliferative capacity, adipogenic differentiation and the expression of the pro-angiogenic VEGF-A protein via activation of the Akt/mTOR/p70S6K signaling pathway [118]. Conversely, SGLT1 downregulation was associated with opposite effects but did not influence glucose intracellular transport, revealing that in basal conditions, SGLT1 is not the main protein involved in glucose uptake. SGLT1 overexpression in perivascular preadipocytes also increased carotid artery wall thickness, leading to remodeling which was associated with enhanced norepinephrine-induced vasoconstriction and depressed sodium nitroprusside-mediated vasodilation. SGLT1 downregulation reversed these detrimental effects [118]. Accordingly, pharmacological inhibition of SGLT1 might mitigate vascular remodeling and dysfunction by acting both on cells in the intima and media as well as on perivascular components [118]. In line with this observation, Forrester et al. reported that DAPA, EMPA, and also mizagliflozin promoted an endothelium-independent vasodilating effect in rat mesenteric artery segments via the NHE1 inhibition-mediated release of calcitonin gene-related peptide from perivascular sensory nerves and the associated indirect activation of the Kv7 potassium channel. Vascular relaxation was low to absent in renal or cardiac septal arteries that, at variance from mesenteric arteries, display a poor perivascular sensory nerve network [151].

The “off-target” vasculoprotective effects of SOTA, the main dual SGTL1/SGLT2 inhibitor, are summarized in Table 2.

**Figure 1:**
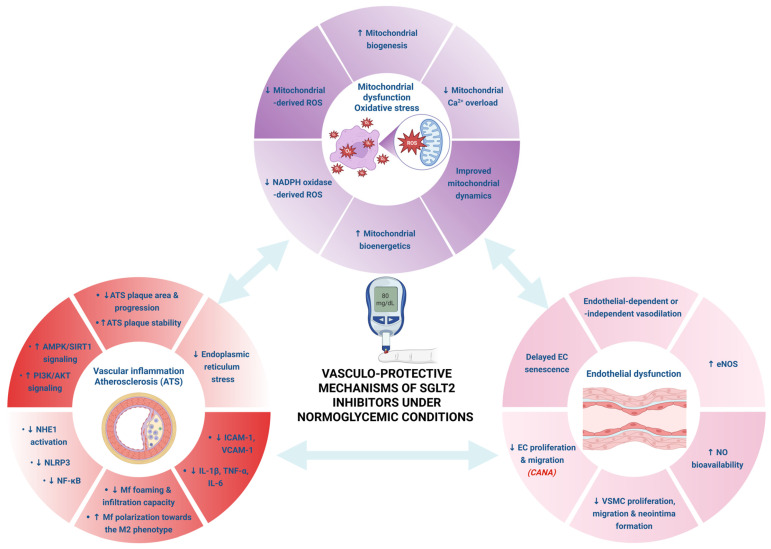
The vasculoprotective mechanisms of SGLT2 inhibitors under normoglycemic conditions. Abbreviations: ↓, decreased; ↑, increased; AKT, Protein kinase B; AMPK, Adenosine monophosphate activated protein kinase; ATS, Atherosclerosis; CANA, Canagliflozin; EC, Endothelial cells; eNOS, Endothelial nitric oxide synthase; ICAM-1, Intercellular Adhesion Molecule 1; IL-1β, Interleukin-1 beta; IL-6, Interleukin 6; Mf, Macrophage; NF-κB, Nuclear factor kappa-light-chain-enhancer of activated B cells; NHE1, Sodium-hydrogen exchanger 1; NLRP3, NLR family pyrin domain containing 3; NO, Nitric oxide; PI3K, Phosphoinositide 3-kinase; ROS, Reactive oxygen species; SIRT1, Sirtuin 1 TNF-α, Tumor necrosis factor alpha; VCAM-1, Vascular cell adhesion protein 1; VSMC, vascular smooth muscle cells. Created in BioRender. Buriman, D. (2026), https://BioRender.com/exrf4l0 (accessed on 5 March 2026).

Figure 1 Summary of the discussed vasculoprotective mechanisms of SGLT2i under normoglycemic conditions.

## 4. Conclusions and Future Perspectives

SGLT2 and SGLT1/2 inhibitors have transformed the therapeutic landscape of cardio-metabolic diseases and have established themselves as potent drugs that possess the ability to achieve cardiovascular protection beyond glycemic control.

One of the primary mechanisms by which SGLT2i confer vascular protection is through the improvement of endothelial function. These inhibitors preserve endothelial function and NO bioavailability, promote vasodilation, and alleviate microvascular dysfunction. Arterial stiffness is a predictor of cardiovascular events and is associated with aging and hypertension. SGLT2i can reduce pulse wave velocity, a measure of arterial stiffness, even in normoglycemic individuals.

Blood pressure reduction due to the diuretic and natriuretic effects of SGLT2i also contributes to their vasculoprotective mechanisms. A lower blood pressure alleviates the mechanical stress on blood vessel walls, reducing the risk of vascular injury and subsequent cardiovascular events.

Inflammation and oxidative stress are key contributors to vascular damage and the progression of cardiovascular disease. SGLT2 inhibitors have been shown to exert anti-inflammatory effects by reducing levels of pro-inflammatory cytokines. Additionally, these inhibitors decrease oxidative stress markers and enhance antioxidant defense systems. These anti-inflammatory and anti-oxidative properties maintain, at least in part, vascular health and slow the progression of atherosclerosis.

However, although sufficient evidence points to their benefits in the context of heart failure, diabetes or kidney damage, large clinical trials are still needed to clarify the long-term therapy outcomes on vascular endpoints such as arterial calcification, carotid disease or peripheral vascular disease both in diabetic—and even more so—in non-diabetic populations, where data are especially lacking. Another issue that must be addressed in the future is their potentially different impacts on various vascular beds which might be of significance for patients with coronary artery disease or pulmonary artery hypertension. Moreover, there is evidence that hormonal factors might influence treatment efficacy, underscoring the importance of systematically addressing sex-related differences both in experimental and clinical settings.

Since the choice between SGLT2 inhibitors or SOTA might be influenced by associated comorbidities like a history of stroke, studies must clarify the patient characteristics that justify their administration. The advantages of gliflozins are clear when the vascular structure or function is threatened by hyperglycemic, hypoxic, pro-inflammatory or pro-oxidant insults. However, in the absence of these deleterious conditions, additional studies are needed to establish the safety and efficacy of SGLT2i in individuals at risk for cardiovascular diseases but without diabetes.

In summary, SGLT2 and SGLT1/2 inhibitors provide multifaceted vascular protection under normoglycemic conditions. Their benefits include restoration of nitric oxide signaling, enhancement of endothelial barrier integrity, suppression of oxidative stress and inflammation, inhibition of maladaptive vascular remodeling and delayed vascular aging, which concurrently contribute to vascular health improvement. Importantly, many of these effects appear to occur independently of glucose lowering and, in some cases, independently of direct SGLT2 inhibition. As such, gliflozins are nowadays emerging as valuable tools in the prevention and management of primary or secondary vascular diseases, extending their therapeutic potential beyond traditional glucose-lowering applications. This expanding role highlights the importance of further basic and clinical research to fully elucidate the pathophysiological mechanisms underlying the vascular protection of SGLT2 and SGLT1/2 inhibitors in the setting of normoglycemia.

## Figures and Tables

**Table 1:** Summary of SGLT2i vasculoprotective effects described in non-diabetic conditions in animal models, cell lines and human samples.

Model	Drug Concentration	Effects	Reference
1. Alleviation of Inflammation			
Macrophages HUVECs	DAPA (0.5 μM) + LPS (20 ng/mL) for 24 h, under normal (5.5 mmol/L) or high glucose (25 mmol/L) conditions	▪ direct anti-inflammatory effects independent of glucose concentration ▪ ↓ TLR-4/NF-κB signaling pathway ▪ ↓ release of pro-inflammatory mediators ▪ ↑ expression of the anti-inflammatory miR-146a ▪ Mf polarization towards the M2 phenotype	[33]
HUVECs	DAPA (1.0–5.0 nM) + TNF-α (10 ng/mL) for 24 h	▪ ↓ ICAM-1 and VCAM-1 protein expression ▪ ↓ PAI-1 mRNA and protein expression (only at high-kdose DAPA—100 nM) ▪ ↓ NF-κB expression	[34]
HAECs	DAPA (1 μM) + TNF-α (100 ng/mL) for 24 h	▪ ↑ PI3K/AKT signaling ▪ ↑ NRF2 expression ▪ partial ↓ of endoplasmic reticulum stress ▪ ↓ NLRP3, NF-κB, IL-1β, IL-6, TNF-α expression ▪ ↓ ICAM-1 and VCAM-1 expression	[38]
Myeloid angiogenic cells and platelets isolated from healthy subjects	DAPA/EMPA (1 to 100 μM) + stearic acid (100 µM) for 16 h	▪ NHE1 inhibition leading to ↓ stearate-induced lipotoxicity ▪ ↓ platelet activation ▪ ↓ myeloid angiogenic cells oxidative stress ▪ ↓ IL-1β, TNF-α release	[40]
Male New Zealand white rabbits fed a 1% high-cholesterol diet	DAPA (1 mg/kg/day) for 8 weeks following balloon catheter injury	▪ ↓ IL-6 and TNF-α release ▪ ↓ TLR-4/NF-κB signaling pathway ▪ ↓ Mf infiltration capacity ▪ ↓ ATS plaque progression	[43]
Platelet-rich plasma or washed platelets obtained from healthy unmedicated adult male and female volunteers	DAPA/EMPA/CANA (1–100 µM—depending on the evaluated endpoint) pre- or co-incubation with sodium nitroprusside and iloprost ± collagen	▪ ↓ human platelet activation	[41]
HAECs	EMPA (0.1–3 µmol/L) for 24 h prior to Ang II (1 μM) for 24 h	▪ ↓ mononuclear leukocyte–endothelial cell interactions ▪ ↓ expression of CCL-2, CCL-5, VCAM-1, ICAM-1 ▪ ↓ p38 MAPK and NF-κB activation	[55]
RAW264.7 cell line	EMPA 1 μM (low-dose group)/5 μM (high-dose group) + ox-LDL 80 ng/mL for 24 h	▪ ↓ macrophage viability and foaming ▪ ↓ IL-6, IL-1β, TNF-α and MCP-1 mRNA expression ▪ ↓ NF-κB protein expression ▪ ↑ AMPK phosphorylation	[51]
RAW264.7 macrophages HASMCs HUVECs	EMPA (30, 50 µM) + ox-LDL (80 μg/mL) for 24 h	▪ ↓ formation of macrophage- and HASMC-derived foam cells ▪ ↑ AMPK phosphorylation with ↑ autophagy in all cell lines ▪ ↓ HASMC proliferation and migration ▪ ↓ IL-6, TNF-α expression in RAW246.7 macrophages and HUVECs	[45]
RAW 264.7 macrophages	EMPA (80 µM) + LPS (200 ng/mL) for 4/48 h	▪ ↓ M1 macrophage polarization ▪ ↓ PGE2, COX-2 and iNOS protein expression ▪ ↓ TNF-α, IL-1β, IL-6, CCL-3, CCL-4, CCL-5, and CXCL10 mRNA expression ▪ ↓ NF-κB, JNK and STAT1/3 phosphorylation via IKKα/β, MKK4/7, and JAK2 signaling	[44]
HAECs (static culture/steady wall shear stress conditions for 24 h)	EMPA (50 μM) + heparinase III (0.5 U/mL for 2 h + 0.1 U/mL for 24 h) + TNF-α (10 ng/mL)	▪ ↓ adhesion of neutrophil-like cells to HAECs after TNF-α treatment and endothelial glycocalyx disruption ▪ ↓ endoplasmic reticulum stress via downregulation of key unfolded protein response genes and ↓ TXNIP mRNA and protein expression	[49]
Porcine coronary artery endothelial cells	EMPA (100 nM, for 30/90 min) before exposure to COVID-19 plasma (3–10% *v*/*v*, for 30 min or 6/24 h)	▪ ↓ SGLT2 expression ▪ ↓ pro-oxidant response in 24 h exposure experiments ▪ ↓ expression of genes associated with cell senescence, thrombosis, proinflammatory cytokines and adhesion molecule production ▪ ↓ NF-κB nuclear translocation ▪ ↑ NO production and endothelial antiaggregatory effect ▪ ↓ endothelial platelet adhesion and thrombin generation	[46]
Male ApoE^−/−^ mice fed a Western diet	EMPA (1 mg/kg/day and 3 mg/kg/day) for 8 weeks	▪ ↓ TNF-α, IL-6, MCP-1, serum amyloid A circulating levels ▪ ↓ area of aortic arch ATS plaques ▪ ↓ inflammatory cell infiltration in ATS plaques	[52]
Male ApoE^−/−^ mice fed a Western diet	EMPA (10 mg/kg/day) for 6 weeks	▪ ↓ area of aortic root ATS plaques ▪ ↓ serum concentration of renin, aldosterone and norepinephrine	[53]
Male ApoE^−/−^ mice fed a high fat diet	EMPA (10 mg/kg/day) for 10 weeks	▪ ↓ area of aortic root ATS plaques with ≈ 50% wider aortic lumen ▪ ↓ VCAM-1 and MCP-1 mRNA at ATS plaque level ▪ ↑ TIMP-1/MMP-2 ratio mRNA level at ATS plaque level	[54]
Male ApoE^−/−^ mice fed a normal chow diet	EMPA (3 mg/kg/day) + Ang II (1000 ng/kg/minute delivered via osmotic minipump) for 28 days	▪ Inhibition of Ang II-induced abdominal aortic aneurysm formation ▪ ↓ medial and adventitial macrophage infiltration in the aortic aneurysms ▪ ↓ neovessel formation ▪ ↓ expression of CCL-2, CCL-5, VEGF, MMP-2, MMP-9 in the aneurysm wall ▪ ↓ p38 MAPK and NF-κB activation in the aneurysm wall	[55]
Male ApoE^−/−^ mice fed a Western diet	EMPA (30 mg/kg/day) for 24 weeks	▪ ↓ area of aortic root ATS plaques ▪ ↓ arterial calcification in the aortic root	[56]
Rat VSMCs Thoracic aorta rings from male C57BL/6 mice	EMPA (1 μM) + osteogenic media (10 mM β-glycerophosphate disodium salt + 3.5 mM CaCl_2_) or inorganic phosphate (2.6 mM) for 5–7 days (VSMCs)/12 days (aortic rings)	▪ ↓ VSMC calcification ▪ ↓ vascular calcification	
Male ApoE^−/−^ mice fed a high-fat diet	EMPA 1.3 mg/kg/day (low-dose group)/3.2 mg/kg/day (high-dose group) for 8 weeks	▪ ↓ area of aortic tree ATS plaques ▪ ↓ Mf infiltration in the ATS plaques ▪ ↓ NF-κB, IL-6, IL-1β protein expression in the ATS plaques ▪ ↓ IL-6 and IL-1β serum concentration	[51]
Male ApoE^−/−^ mice fed a high-fat diet	EMPA 1.5 mg/kg/day (low-dose group)/3.5 mg/kg/day (high-dose group) for 8 weeks	▪ ↓ area of abdominal/thoracic aorta and aortic arch ATS plaques	[45]
HCAECs	CANA (3, 10 μM) or EMPA (1, 3, 10 μM) or DAPA (0.5, 3, 10 μM) for 16 h prior to LPS (1 μg/mL) for 3 h	▪ ↓ IL-6 release and ERK 1/2 phosphorylation via the ↓ of hexokinase II expression (CANA) ▪ ↑ AMPK activation (CANA) ▪ Effects present for CANA, but not EMPA or DAPA	[58]
HUVECs HAECs	CANA (10 μmol/L) or EMPA (1 μmol/L) or DAPA (1 μmol/L) for 15 min prior to IL-1β (10 ng/mL) for 6 or 24 h	▪ ↓ adhesion of pro-monocytic U937 cells ▪ ↓ IL-6 and MCP-1 secretion ▪ ↑ AMPK phosphorylation ▪ Effects present for CANA, but not EMPA or DAPA	[59]
Primary bone marrow-derived macrophages	CANA (10μM) for 90 min + addition of LPS (10 ng/mL) for 24 h	▪ ↑ AMPK and acetyl-CoA carboxylase phosphorylation ▪ ↓ STAT-1 (marker of M1 polarization) and TNF-α secretion independently of AMPK ▪ ↓ IL-1β secretion in an AMPK-dependent manner	[63]
RAW264.7 macrophages THP-1 cells	LPS (1 µg/mL) for 12 h + addition of CANA (40 µM) for 3–12 h	▪ ↓ IL-1α, IL-6 and TNF-α release ▪ ↓ ROS release ▪ ↑ p62 mRNA and protein expression, contributing to IL-1α and TNF-α degradation ▪ ↑ AMPK phosphorylation and cell autophagy	[57]
Male ApoE^−/−^ mice fed a high-fat diet	CANA (10 mg/kg/day) for 5 weeks	▪ ↓ area of aortic root ATS plaques ▪ ↓ MCP-1 and VCAM-1 expression in the aortic root ▪ ↑ collagen content and TIMP-1/MMP-2 ratio in the ATS plaques	[61]
Male ApoE^−/−^ mice fed a Western diet	CANA (20 mg/kg/day) for 15 weeks	▪ ↓ area of aortic root ATS plaques ▪ ↓ Mf infiltration in the ATS plaques ▪ ↓ IL-1β, IL-6, TNF-α serum level ▪ ↑ collagen fiber content in ATS plaques and aortic autophagy	[62]
Female ApoE^−/−^ mice fed a Western diet	CANA (30 mg/kg/day) for 6 weeks	▪ No effect on the area of ATS plaques ▪ ↓ IL-1β serum level	[63]
Male NIH mice fed standard chow diet	CANA (50 mg/kg/day) for 3 days + LPS (2 mg/kg) 4 h prior to euthanasia	▪ ↓ IL-6, TNF-α serum level	[57]
Rat VSMCs Rat thoracic aorta rings Human tibial artery rings	CANA (5, 10, 20 μM) + calcifying medium (10 mM β-glycerophosphate + 3 mM CaCl_2_) for 7 days	▪ ↓ rat and human arterial ring calcification ▪ ↓ VSMC calcification ▪ ↓ VSMC osteogenic differentiation via ↓ NF-κB/NLRP3 signaling ▪ ↓ VSMC apoptosis	[64]
Male Sprague Dawley rats with CKD (5/6 nephrectomy model) fed a high-calcium-and-phosphorus diet	CANA (10 mg/kg/day) for 3 weeks	▪ ↓ aortic calcification ▪ ↑ α-SMA (contractile marker) with ↓ osteogenic transition in aortas	
C57BL/6J mice with Vitamin D3 overload	Vitamin D3 (5 × 10^5^ IU/kg/day) + CANA (5 or 10 mg/kg/day) for 3 days	▪ ↓ aortic calcification ▪ ↑ α-SMA (contractile marker) with ↓ osteogenic transition in aortas	
**2. Protection of Mitochondrial Structure/Function and Alleviation of Oxidative Stress**			
HCAECs	DAPA (10 μM) for 24 h prior to H/R injury	▪ ↑ average mitochondrial length ▪ ↓ mitochondrial fission and ↑ fusion	[73]
Mouse cardiac endothelial cells	DAPA (100 nM or 1 μM) + cobalt chloride (100 μM) for 24 h	▪ ↑ mitochondrial respiration but no effect on the rate of glycolysis ▪ ↑ intracellular ATP, total adenine nucleotide pool and ATP/ADP ratio (DAPA 1 μM) ▪ ↑ NO production	[75]
Female C57BL/6 mice	DAPA (35 mg/kg/day) for 6 weeks	▪ ↑ NO production ▪ ↑ coronary capillary density	
HUVECs	ox-LDL (100 μg/mL) for 48 h + DAPA (10 μM) for 4 h	▪ ↓ ferroptosis ▪ ↑ NRF2, PGC-1α, mtTFA protein expression and NRF2 nuclear translocation suggesting ↑ mitochondrial biogenesis ▪ ↑ mitochondrial respiration, ATP production, mtDNA copy number and mitochondrial biogenesis ▪ ↓ mitochondrial ROS production ▪ ↑ RAP1B protein expression, responsible for the beneficial mechanisms listed above	[74]
ApoE^−/−^ mice (±RAP1B^−/−^ knockout) fed a high-fat diet	DAPA (10 mg/kg/day) for 6 weeks	▪ RAP1B-dependent: ○ ↓ area of thoracic aorta ATS plaques ○ ↑ GPX4, NRF2 and PGC-1α artery levels ○ ↓ ferroptosis ○ ↓ oxidative stress (↑ SOD, GSH/GSSG ratio) ○ ↑ mitochondrial biogenesis and OXPHOS ▪ RAP1B-independent ↑ ATS plaque stability	
Obese male mice fed a high-fat diet	DAPA (1 mg/kg/day) for 16 weeks	▪ ↑ CD31 mRNA expression ▪ ↓ vascular endothelial dysfunction	[76]
HUVECs	DAPA (1 μM) + palmitic acid (200 μM) for 24 h	▪ ↑ angiogenic ability ▪ ↓ apoptosis ▪ ↑ mitochondrial membrane potential, viability and ATP production ▪ ↓ palmitic acid-induced mitochondrial swelling and structural degradation ▪ improved mitochondrial biogenesis ▪ ↑ SIRT1/PGC-1α pathway activation, responsible for the mito-protective effects	
HCAECs HUVECs	EMPA or DAPA (1 µM) preincubation for 2 h + coincubation with TNF-α for 24 h	▪ ↓ ROS generation ▪ ↑ NO bioavailability ▪ No effect on TNF-α-induced disruption of eNOS signaling, cellular hyperpermeability or increased ICAM-1 and VCAM-1 expression	[72]
HUVECs	EMPA (1 µM) + high glucose (30 mmol/L) for 24 h	▪ ↓ mitochondrial Ca^2+^ overload ▪ ↓ ROS production	[68]
Human brain microvascular endothelial cells	EMPA (1 µM) + high glucose (30 mmol/L) for 48 h EMPA (1 µM) preincubation for 24 h + H_2_O_2_ (0.5, 1, 5 mM) for 5 h	▪ ↓ high glucose-induced endothelial hyperpermeability ▪ ↑ cell viability after exposure to H_2_O_2_	
HCAECs	EMPA (1 µM) or DAPA (1 µM) or CANA (3 µM) for 2 h + 10% stretch for 24 h	▪ ↓ endothelial hyperpermeability ▪ ↓ ROS production, most likely via NHE1 and NOX inhibition ▪ No effect on IL-6 and IL-8 secretion	[77]
HCAECs	EMPA (1 µM) for 2 h + 10% stretch for 24 h	▪ ↓ NOX activity via inhibition of the NHE/Na^+^/NCX/Ca^2+^/PKC axis ▪ ↓ ROS generation	[78]
HCAECs (laminar shear stress conditions for 6 h)	EMPA (1 µM) for 2 h + TNF-α (10 ng/mL) for 6 h	▪ ↓ ROS generation via inhibition of NHE1 and of cellular Ca^2+^ entry ▪ ↑ NO bioavailability but no effect on eNOS expression or phosphorylation ▪ No effect on IL-6, IL-8, MCP-1, ICAM-1 and VCAM-1 expression	[79]
HCAECs HUVECs	EMPA (1 µM) + TNF-α (10 ng/mL) for 6 h	▪ ↓ ROS generation via inhibition of NHE1 and lowering intracellular Na^+^	[80]
Human internal mammary artery rings from overweight CAD patients	EMPA (10 µM) + high glucose (400 mg/dL) or Ang II (100 nM) for 12 h	▪ ↓ MAO-A and -B expression ▪ ↓ ROS generation ▪ ↑ endothelium-dependent relaxation	[83]
Male endothelial-specific *AMPKα1*-knockout/*FUNDC1*-knockout mice.	EMPA (10 mg/kg/day) for 7 days prior to I/R injury	▪ ↓ endothelial swelling, luminal stenosis and microvascular hyperpermeability	[81]
CMECs	Cells isolated from the left ventricle of the EMPA-treated mice undergoing I/R	▪ ↑ eNOS phosphorylation ▪ ↓ endothelin 1 and ICAM-1 expression ▪ ↓ mitochondrial fission and ↑ fusion ▪ ↓ mitochondrial ROS formation ▪ Stabilized mitochondrial membrane potential with ↓ mPTP opening rate and ↓ caspase-9 activation ▪ FUNDC1-dependent mitophagy through the AMPKα1/ULK1 pathway ▪ ↑ GSH, SOD, GPX levels	
Coronary artery VSMC	EMPA (500 nM) for 24 h	▪ ↓ filamentous actin and phosphorylated (inactive) cofilin, partially mediating the EMPA-related de-stiffening effect	[82]
Aged (80-week-old) male C57BL/6 J mouse mesenteric artery rings	EMPA (14 mg/kg/day) for 6 weeks	▪ ↑ endothelium-dependent vasodilation ▪ ↑ eNOS activation ▪ ↑ nitric oxide synthase activation ▪ ↓ arterial stiffness ▪ ↓ XO expression ▪ ↓ pathways involved in ROS generation ▪ ↓ filamentous actin and phosphorylated cofilin ▪ No impact on mitochondrial OXPHOS	
HUVECs	CANA (0.1–0.5 μM) + palmitic acid (0.3 mM) for 24 h	▪ ↓ palmitic acid-induced cell cycle arrest ▪ ↓ intracellular ROS and lipid peroxidation ▪ ↓ ROS production, inhibiting ERK activity ▪ ↓ ROS/ERK signaling, inhibiting ferroptosis and delaying cell aging	[85]
HUVECs	CANA (10 µM and 100 µM) or DAPA (0.3 µM and 3 µM) or EMPA (100 µM) for 2–3 h	▪ CANA (at supra-pharmacological and, partially, pharmacological concentrations) ○ ↓ mitochondrial respiration (primarily complex I) ○ ↓ glycolysis rate ○ ↓ beta-oxidation ▪ No effect of other SGLT2i on mitochondrial respiration or glycolysis	[87]
Male ApoE^−/−^ mice fed a Western diet	CANA (20 mg/kg/day) for 15 weeks	▪ ↓ ROS ▪ ↑ serum levels of GSH-PX and SOD ▪ ↓ NOX4 mRNA ▪ ↑ NRF2 and GST mRNA ▪ ↑ eNOS mRNA	[62]
**3. Improvement of Endothelial Function**			
HAECs	DAPA (1 μM) + TNF-α (100 ng/mL) for 24 h	▪ ↑ eNOS mRNA expression levels ▪ ↑ NO bioavailability	[38]
C57BL/6 J mouse aortic rings	DAPA (100 μM and 300 μM)—acute administration in organ bath	▪ ↑ direct vasorelaxation	[92]
HUVECs	H_2_O_2_ (100 µM) for 1 h + DAPA (10 µM) for 3 days	▪ ↓ intracellular ROS and peroxynitrite accumulation ▪ ↑ NO bioavailability ▪ ↑ eNOS activation ▪ delayed cellular senescence ▪ ↓ endothelial dysfunction via SIRT1 activation	
Hypertensive male Dahl salt-sensitive rats fed a high-salt diet	DAPA (0.1 mg/kg/day) for 6 weeks	▪ ↓ VCAM-1 and E-selectin expression ▪ ↑ eNOS expression	[93]
HUVECs	DAPA (1 μM) for 10 min (ammonium pulse technique)	▪ ↓ NHE1 activity	
HCAECs	DAPA (10 μM) for 24 h prior to H/R injury	▪ ↑ viability, proliferation ▪ ↑ eNOS activity and VEGF expression ▪ ↓ endothelin 1 protein expression ▪ ↓ cellular hyperpermeability ▪ ↓ apoptosis and preserved cytoskeletal integrity via XO-induced SERCA2 inactivation leading to normalized intracellular calcium balance with CaMKII/cofilin pathway regulation	[73]
Endothelial-specific SERCA2 knockout mice	DAPA (40 mg/kg/day) for 7 days prior to I/R injury via left anterior descending coronary artery ligation	▪ ↓ I/R injury-induced vascular structural changes (i.e., swollen endothelial cells with DNA fragmentation) ▪ ↓ ICAM-1, IL-6, MCP1 and TNFα expression ▪ ↓ endothelial cell apoptosis	
Male C57BL/6J mouse abdominal aortic rings	DAPA (1 nM–10 µM)—acute administration in organ bath	▪ direct endothelium-independent vasorelaxation	[34]
Aortic rings from ApoE^−/−^ adult and aged mice fed a high-fat diet	DAPA (1 mg/kg/day) for 4 weeks	▪ ↑ endothelium-dependent vasorelaxation ▪ ↓ NF-κB activation ▪ ↓ ICAM-1 expression ▪ ↓ Mf infiltration	
Male New Zealand white rabbit thoracic aorta rings	DAPA (100 μM and 300 μM)—acute administration in organ bath	▪ ↑ endothelium-independent vasorelaxation mediated by activation of the VSMC potassium Kv1.5 channel and PKG	[94]
Male Sprague Dawley rat small mesenteric artery rings	DAPA (0.001–100 μM)—acute administration in organ bath	▪ ↑ endothelium-independent vasodilation via VSMC Kv7 potassium channel activation ▪ Vascular effects independent of NO-sGC-PKG signaling axis or SGLT2 inhibition	[95]
Male Sprague Dawley rat left descending coronary artery rings	DAPA (1–500 μM)—acute administration in organ bath	▪ ↑ endothelium-independent vasodilation via inhibition of extracellular Ca^2+^ influx ▪ Vascular effects independent of the NO/cGMP pathway, potassium channels or prostacyclin	[96]
Aortic VSMCs	DAPA (50 μM)	▪ ↓ phosphorylation of the 20 kDa myosin light chain	
Neurogenic hypertensive Schlager (BPH/2J) mice fed a high-fat diet	DAPA (40 mg/kg every 2 days) for 2 weeks	▪ ↑ endothelium-dependent relaxation via ↓ asymmetric dimethylarginine (endogenous NOS inhibitor)	[97]
Coculture of human CMECs and adult rat cardiomyocytes	EMPA (1 μM) + TNF-α (10 ng/mL)/IL-1β (10 ng/mL) for 6 h	▪ EMPA supports the beneficial effects of CMECs on cardiomyocyte contractility and relaxation by enhancing NO bioavailability ▪ ↑ NO bioavailability in CMECs via ↓ mitochondrial and cytoplasmic ROS level	[99]
Human coronary artery VSMCs	EMPA (750 nM) for 24 h	▪ Cytostatic effect with migratory capacity inhibition	[7]
Human coronary artery endothelial cells	EMPA (750 nM) for 24 h	▪ No effect on proliferation or migratory capacity	
Male C57BL/6 mice	EMPA (10 mg/kg/day) for 7 days	▪ ↓ neointima formation after wire-induced injury of the femoral artery ▪ No effect on re-endothelialization after electrical de-endothelialization of the carotid artery	
Coculture of human CMECs and adult rat cardiomyocytes	EMPA (1 μM) + endothelial growth medium-2MV supplemented with 15% human uremic serum	▪ EMPA reverses the uremic serum–induced loss of NO bioavailability in CMECs, thus supporting cardiomyocyte function ▪ ↑ NO bioavailability in CMECs via ↓ mitochondrial and cytoplasmic ROS level	[100]
Human microvascular endothelial cells	EMPA (500 nM) for 24 h prior to H/R and during the 3 h hypoxia period + STAT-3 inhibitor (Stattic, 500 nM) during reoxygenation	▪ ↑ cell viability via STAT-3 activation ▪ ↓ ROS production independently of STAT-3 activation	[101]
C57BL/6 male mice	EMPA (10 mg/kg/day) for 6 weeks prior to I/R injury via left anterior descending coronary artery ligation	▪ ↓ infarct size and myocardial oxidative stress ▪ ↑ myocardial VEGF and SOD2 levels ▪ ↑ survival of endothelial cells via STAT-3 activation	
Human iPSC-derived endothelial cells isolated from human subjects carrying the ALDH2*2 allele	EMPA (5 μM) for 1 day prior to ethanol (5 mM) coincubation for 1 day	▪ ↓ ROS generation and ↑ NO production via inhibition of NHE1 and activation of the AKT/eNOS pathway ▪ ↓ monocyte adhesion ▪ ↑ endothelial cell tube formation	[102]
Aortic rings from ALDH2*1/*2 knock-in mice	EMPA (10 mg/kg/day—delivered via osmotic pump) + ethanol intraperitoneal injection (20%, 1 g/kg/day) for 21 days	▪ limited vascular remodeling ▪ ↓ aorta area and wall thickness ▪ ↑ endothelium-dependent relaxation	
Male New Zealand white rabbit thoracic aorta rings	EMPA (30, 100, 300, and 1000 μM)—acute administration in organ bath	▪ ↑ endothelium-independent vasodilation mediated by PKG signaling and the activation of Kv, but not KATP, BKCa, or Kir channels ▪ ↑ PKG-1 expression in aortic smooth muscle layer	[104]
Male Sprague Dawley rat resistance-size mesenteric arteries	EMPA (0.001–100 µM)—acute administration in organ bath	▪ ↑ endothelium-independent vasodilation via VSMC Kv1.5 and Kv7, but not KATP or BKCa channel activation ▪ Vascular effects independent of NO-sGC-PKG signaling axis or endothelial prostacyclin	[105]
Adult male Wistar rats	EMPA (30 mg/kg/day) for 18 days, beginning 4 days before carotid artery balloon injury	▪ ↓ neointima formation in carotid arteries ▪ ↓ injury-induced upregulation of PDGF-related proteins (phosphorylated Akt, phosphorylated STAT3, phosphorylated ERK)	[103]
Rat aortic VSMCs	EMPA (0.1–10 μmol/L) + PDGF-BB (60 ng/mL) for 24 h	▪ ↓ PDGF-BB-mediated proliferation, migration and apoptosis ▪ ↓ PDGF-related signaling (↓ phosphorylation of PDGF receptor β, Akt, STAT3 and ERK) ▪ Effects independent of SGLT2 inhibition	
Female Yorkshire pigs	EMPA (10 mg/day) for 2 months after left anterior descending coronary artery occlusion (model of HFrEF)	▪ ↑ eNOS phosphorylation ▪ ↑ NO bioavailability ▪ ↑ eNOS/NO/cGMP/PKG/titin pathway activity with improved cardiac diastolic function	[117]
Male wild-type mice with a C57BL/6NCrSlc background	EMPA (0.03% *w*/*w*) in normal chow for 2 weeks after transverse aortic constriction (left ventricular pressure overload model)	▪ ↓ endothelial apoptosis and capillary rarefaction ▪ ↑ eNOS phosphorylation and NO production ▪ ↓ ROS production in endothelial cells ▪ ↑ 3-hydroxybutyrate myocardial and plasma levels	[106]
HUVECs	3-hydroxybutyrate (10 mM) for 1 h prior to norepinephrine (10 ng/mL) coincubation for 72 h	▪ ↓ catecholamine-induced downregulation of the AKT/eNOS/NO pathway via 3-hydroxybutyrate increase and ROS level reduction	
Male Wistar rats	EMPA (30 mg/kg/day) for 5 weeks, initiated 1 week prior to Ang II (0.4 mg/kg/day) treatment administered via osmotic mini-pumps for 4 weeks	▪ ↓ ROS generation in the arterial wall ▪ ↓ nitrotyrosine level with improved aortic eNOS/ROS balance ▪ ↓ ACE and AT1R expression ▪ ↓ NOX1 and ↑ NOX4 mRNA levels ▪ ↓ VCAM-1, ICAM-1, MCP-1, MMP-2, MMP-9 mRNA expression ▪ ↓ SGLT1 protein expression at thoracic aorta, mesenteric and coronary micro-vessel levels ▪ ↓ SGLT2 protein expression at thoracic aorta level	[107]
HUVECs	CANA (1–50 µM) for 24 h	▪ ↑ HO-1 expression ▪ ↓ endothelial DNA synthesis, proliferation and migration capacity, independently of heme oxygenase-1	[113]
	CANA (1–50 µM) + TNF-α (10 ng/mL) + high glucose (25 mM) for 24 h	▪ ↓ monocyte adhesion via heme oxygenase-1-mediated mechanisms	
HUVECs	CANA (1–50 μM) or EMPA (1–50 μM) or DAPA (1–50 μM) for 3 days	▪ ↓ DNA synthesis and cell proliferation by clinically relevant concentrations of CANA (but only very high concentrations of EMPA and DAPA) ▪ ↓ migration capacity by high concentrations of CANA and EMPA ▪ blocked cell cycle in the G_0_/G_1_ phase by CANA, via ↓ cyclin A expression ▪ ↓ endothelial tube formation by CANA, but not EMPA or DAPA	[114]
Male C57BL/6 mouse aortic rings	CANA (10, 20, 50 μM) + VEGF-A_164_ (10 ng/mL) for 5 days	▪ ↓ sprouting of endothelial cell capillaries from mouse aortic rings	
Male Sprague Dawley rat resistance-size mesenteric arteries	CANA (0.001–100 μM) acute administration in organ bath	▪ ↑ endothelium-independent vasodilation via smooth muscle cell Kv1.5, Kv7 and Kv2.1 potassium channel activation ▪ Vascular effects independent of SGLT2 inhibition, SERCA pump activation or NO-sGC-PKG and prostacyclin signaling	[109]
Male Sprague Dawley rats	CANA (4 mg/kg) single dose	▪ ↓ systolic and diastolic blood pressure	
Male Sprague Dawley rat thoracic aorta segments	CANA (10 µM) preincubation for 30 min before organ bath experiments	▪ ↑ endothelium-dependent relaxation	[110]
Male Wistar rat thoracic aorta rings	CANA (50 µM) for 24 h in nitrogen-gassed saline (pO_2_ 70–74 mmHg) to mimic vascular I/R injury	▪ ↑ endothelium-dependent relaxation ▪ ↓ ICAM-1 and nitrotyrosine and ↑ PECAM-1 expression ▪ downregulation of IL-1a, IL-6 and NADPH oxidase organizer 1 genes ▪ ↓ I/R-induced upregulation of CD40	[111]
Male C57BL/6 mouse small coronary artery or pulmonary artery rings	CANA (10 µmol/L) preincubation for 20 min before organ bath experiments	▪ ↓ endothelial-independent vasodilation in pulmonary, but not coronary, arteries	[112]
Human pulmonary artery smooth muscle cells	CANA (10 µmol/L) preincubation	▪ ↓ sodium nitroprusside-induced membrane hyperpolarization and K^+^ channel activation, possibly responsible for the vasodilation inhibition observed in the rodent model	
Rat and human aortic VSMCs	CANA (1–50 μM) for 4 days or EMPA (1–50 μM) or DAPA (1–50 μM) for 3 days	▪ ↓ cell proliferation by clinically relevant concentrations of CANA (but only very high concentrations of EMPA and DAPA) ▪ blocked cell cycle in the G_0_/G_1_ phase and ↓ DNA synthesis by CANA ▪ ↓ migration capacity by CANA ▪ ↑ HO-1 expression and activity by CANA (but not EMPA or DAPA) via ROS-NRF2 pathway activation	[115]
Human visceral adipose tissue artery rings obtained from obese and non-obese individuals	CANA (10^−6.5^–10^−4^ mol/L)—acute administration in organ bath	▪ ↑ endothelium-independent vasodilation, slightly more potent in the obese population and possibly mediated by NHE1 inhibition	[116]

Abbreviations: ↓, decreased; ↑, increased; ACE, angiotensin-converting enzyme; AKT, Protein kinase B; AMPK, Adenosine monophosphate activated protein kinase; Ang II, Angiotensin II; AT1R, angiotensin type 1 receptor; ATS, Atherosclerosis; CAD, coronary artery disease; CaMKII, Calcium/Calmodulin (CaM)-dependent kinase II; CANA, Canagliflozin; CCL-2, Chemokine (C-C motif) ligand 2; CCL-3, Chemokine (C-C motif) ligand 3; CCL-4, Chemokine (C-C motif) ligand 4; CCL-5, Chemokine (C-C motif) ligand 5; cGMP, Cyclic guanosine monophosphate; CKD, Chronic kidney disease; CMECs, Cardiac microvascular endothelial cells; CXCL10, C-X-C motif chemokine ligand 10; DAPA, Dapagliflozin; EMPA, Empagliflozin; eNOS, Endothelial nitric oxide synthase; ERK 1/2, Extracellular signal-regulated kinases; FUNDC1, FUN14 domain-containing 1; GPX4, Glutathione peroxidase 4; GSH/GSSG, Oxidized/Reduced Glutathione Ratio; GSH-PX, Glutathione peroxidase; GST, Glutathione S-transferases; HAECs, Human aortic endothelial cells; HASMCs, Human aortic smooth muscle cells; HCAECs, Human coronary artery endothelial cells; HFrEF, heart failure with reduced ejection fraction; HO-1, Heme oxygenase-1; H/R, Hypoxia/reoxygenation; HUVECs, Human umbilical vein endothelial cells; ICAM-1, Intercellular Adhesion Molecule 1; IKK, Inhibitor of nuclear factor-κB (IκB) kinase; IL-1β, Interleukin-1 beta; IL-6, Interleukin 6; iPSC, induced pluripotent stem cell; I/R, ischemia/reperfusion; JAK2, Janus kinase 2; JNK, c-Jun N-terminal kinases; LPS, Lipopolysaccharide; MAO, Monoamine oxidase; p38 MAPK, p38 mitogen-activated protein kinase; MCP-1, Monocyte chemoattractant protein-1; Mf, Macrophage; MKK, Mitogen-activated protein kinase kinase; MMP-2, Matrix metalloproteinase-2; MMP-9, Matrix metalloproteinase-9; mPTP, Mitochondrial permeability transition pore; mRNA, Messenger ribonucleic acid; mtTFA, Mitochondrial transcription factor A; NCX, Sodium calcium exchanger; NF-κB, Nuclear factor kappa-light-chain-enhancer of activated B cells; NHE1, Sodium-hydrogen exchanger 1; NLRP3, NLR family pyrin domain containing 3; NO, Nitric oxide; NOXs, NADPH oxidase; NRF2, Nuclear factor erythroid 2-related factor 2; ox-LDL, Oxidized low-density lipoprotein; OXPHOS, oxidative phosphorylation; PAI-1, Plasminogen activator inhibitor type 1; PDGF-BB, Platelet-Derived Growth Factor subunit BB; PECAM-1, Platelet endothelial cell adhesion molecule 1; PGC-1α, Peroxisome proliferator receptor gamma coactivator 1-alpha; PI3K, Phosphoinositide 3-kinase; PKC, Protein kinase C; PKG, Protein kinase G; ROS, Reactive oxygen species; SERCA2, Sarco(endo)plasmic reticulum calcium-ATPase 2; sGC, soluble guanylate cyclase; SIRT1, Sirtuin 1; SOD, Superoxide dismutase; STAT, Signal transducer and activator of transcription; α-SMA, α-smooth muscle actin; TIMP-1, Tissue inhibitor of metalloproteinases-1; TNF-α, Tumor necrosis factor alpha; TLR-4, Toll-like receptor 4; TXNIP, Thioredoxin interacting protein; ULK1, Unc-51-like autophagy activating kinase 1; VCAM-1, Vascular cell adhesion protein 1; VEGF, Vascular endothelial growth factor; VSMCs, Vascular smooth muscle cells; XO, xanthine oxidase.

**Table 2:** Summary of SOTA vasculoprotective effects described in non-diabetic conditions in animal models, cell lines and human samples.

Model	Drug Concentration	Effects	Reference
Porcine coronary artery EC	SOTA (100 nmol/L) for 30 min + H_2_O_2_ (100 μmol/L) for 24 h	▪ ↓ basal glucose uptake ▪ ↓ H_2_O_2_-induced glucose uptake	[125]
HUVECs	SOTA (100 nM) for 30 min + Ang II (100 nM) for 24 h	▪ ↓ expression of SGLT2 ▪ ↓ expression of the p22phox protein, caveolin-1 and proteins linked to the AKT pathway	[131]
HUVECs	SOTA (100 nM) for 30 min + IL-6 (12 ng/mL) or LPS (100 ng/mL) for 24 h	▪ ↑ expression of PRDX 5 and 6 and GPX-1 in response to IL-6 ▪ ↓ expression of PRDX 1 and GPX-1 and ↑ expression of thioredoxin in response to LPS	[132]
Male Wistar rat aortic arch and thoracic aorta segments	SOTA (100 nM) for 30 min + Ang II (100 nM) for 15 h	▪ ↓ expression of SGLT1 and SGLT2 ▪ ↓ upregulation of VCAM-1 ▪ ↓ pro-oxidant response	[121]
Porcine coronary artery EC	SOTA (100 nM) for 30 min + Ang II (100 nM—for 30 min or 24 h) or CAD-MPs (10 nM PhtdSer eq for 48 h)	▪ ↓ expression of SGLT1 and SGLT2 ▪ ↓ endothelial pro-oxidant response (long-term Ang II exposure experiments lasting 24 h) ▪ ↓ ACE/AT1R/NADPH oxidase pathway activation ▪ ↓ Ang II-induced pro-senescence response ▪ ↓ downregulation of eNOS and NO formation ▪ ↓ upregulation of VCAM-1, MCP-1 and tissue factor	
Male Wistar rat thoracic aorta and secondary branch mesenteric artery segments	Ang II (0.4 mg/kg/day) administered in vivo via osmotic mini-pumps for 4 weeks + in vitro SOTA (100 nM) treatment for 30 min	▪ ↓ endothelial pro-oxidant response	[107]
Internal thoracic artery segments harvested via bypass surgery from patients with CAD	SOTA (100 nmol/L)	▪ ↓ vascular oxidative stress	[134]
Porcine coronary artery EC	SOTA (100 nM) for 30 min + TNF-α (10 ng/mL) for 24 h	▪ ↓ expression of VCAM-1, ACE1 and AT1R ▪ ↑ expression of eNOS protein levels ▪ ↓ endothelial glucose uptake ▪ ↓ NF-κB activation ▪ ↓ the pro-oxidant response ▪ preserved NO formation in response to bradykinin	
HAAECs	SOTA (100 μM) for 18 h prior to Tunicamycin (1 μg/mL) for 24 h	▪ ↓ expression of endoplasmic reticulum stress markers TXNIP and NLRP3	[135]
Sprague Dawley rats with chemically induced PAH	Monocrotaline (40 mg/kg) + CANA (30 mg/kg/day) for 4 weeks	▪ improvement of echocardiographic parameters ▪ ↓ thickness of the pulmonary artery wall ▪ ↓ vascular inflammatory cell infiltration and muscle cell activity ▪ ↓ expression of SGLT1 and PCNA ▪ ↑ AMPK activation	[124]
Rat PASMCs	PDGF-BB (20 ng/mL) + CANA (20 μM) for 48 h	▪ anti-proliferative effect ▪ ↓ expression of PCNA ▪ ↑ AMPK activation via SGLT1 modulation	
Human PASMCs	Hypoxia (3% O_2_) + CANA (20 μM) for 48 h		
HUVECs	Hypoxia (100% N_2_) + Phlorizin (500 μM) for 2 h	▪ ↓ intracellular Ca^2+^ level ▪ ↓ activation of phospholipase A2	[145]
C57BL/6J male mice	Phlorizin (460 μg/μL) initiated 7 days before asymmetric common carotid artery surgery and maintained 35 days thereafter	▪ ↑ cerebral blood flow ▪ ↓ SGLT1, IL-1β and TNF-α expression in the brain	[146]
BBMECs	Hypoxia (95% N_2_ and 5% CO_2_) + Phlorizin (50 μM) for 12 h	▪ ↓ glucose uptake	[147]
CD-1 mice	Middle cerebral artery occlusion for 6 h + Phlorizin (200 mg/kg body mass) administered 1 h after focal ischemia induction	▪ ↓ glucose blood-to-brain transport ▪ ↓ brain infarct and edema areas	
Male Sprague Dawley rats fed with high-fat chow to induce the experimental model of carotid atherosclerosis	Phlorizin-Liposomes (20 mg/kg) administered by gavage for 4 weeks	▪ ↓ vascular inflammatory and foam cell infiltration ▪ ↓ carotid lipid deposition ▪ improved endothelial structure ▪ ↓ expression of phosphorylated NF-κB, IL-1β, TNF-α, COX-2 and iNOS ▪ ↑ expression of Nrf2 and of its downstream antioxidant enzymes NAD(P)H quinone oxidoreductase 1, heme oxygenase 1 and GCLC.	[148]
Activated human CD4^+^ T cells	Phlorizin (25 or 100 μmol/L) or EMPA (0.5 μmol/L) in normal (5.6 mmol/L) or high (16.7 mmol/L) glucose concentration culture medium	▪ ↓ glucose uptake (both SGLT inhibitors) ▪ ↓ release of interferon-γ (Phlorizin at both glucose concentrations, EMPA at high glucose concentration)	[150]
Perivascular preadipocytes from male C57BL/6J mice	Lentiviral vector used to knockdown the SGLT1 gene	▪ ↑ cell apoptosis ▪ ↓ cell proliferation and adipogenic differentiation ▪ ↓ expression of VEGF-A protein ▪ ↓ carotid artery wall thickness and remodeling ▪ ↓ norepinephrine-induced vasoconstriction ▪ ↑ sodium nitroprusside-induced vasodilation	[118]
Second-order mesenteric and cardiac septal resistance artery and conduit renal artery segments from male rats	DAPA, EMPA or mizagliflozin applied cumulatively (1–100 μM)	▪ ↑ endothelium-independent vasodilation in rat mesenteric artery segments via the NHE1 inhibition-mediated release of calcitonin gene-related peptide from perivascular sensory nerves ▪ indirect activation of the Kv7 potassium channel ▪ no effect on renal or cardiac septal arteries	[151]

Abbreviations: ↓, decreased; ↑, increased; ACE1, Angiotensin-converting enzyme 1; AKT, Protein kinase B; AMPK, Adenosine monophosphate activated protein kinase; Ang II, Angiotensin II; AT1R, Angiotensin II receptor type 1; BBMECs, Bovine brain microvascular endothelial cells; CAD-MPs, Blood-derived microparticles collected from coronary artery disease patients; DAPA, Dapagliflozin; ECs, Endothelial cells; EMPA, Empagliflozin; eNOS, Endothelial nitric oxide synthase; GCLC, Glutamate-cysteine ligase catalytic subunit; GPX-1, Glutathione peroxidase-1; HAAECs, Human abdominal aortic endothelial cells; HUVECs, Human umbilical vein endothelial cell; IL-6, Interleukin 6; LPS, Lipopolysaccharide; MCP-1, Monocyte chemoattractant protein-1; NF-κB, Nuclear factor kappa-light-chain-enhancer of activated B cells; NHE1, Sodium-hydrogen exchanger 1; NLRP3, NLR family pyrin domain containing 3; NO, Nitric oxide; PAH, Pulmonary artery hypertension; PASMCs, Pulmonary artery smooth muscle cells; PCNA, Proliferating cell nuclear antigen; PDGF-BB, Platelet-derived growth factor-BB; PRDX, Peroxiredoxin; SOTA, Sotagliflozin; TXNIP, Thioredoxin interacting protein; VCAM-1, Vascular cell adhesion protein 1; VEGF-A, Vascular endothelial growth factor-A.

## Data Availability

No new data were created or analyzed in this study. Data sharing is not applicable to this article.
